# The Anaerobically Induced sRNA PaiI Affects Denitrification in *Pseudomonas aeruginosa* PA14

**DOI:** 10.3389/fmicb.2017.02312

**Published:** 2017-11-23

**Authors:** Muralidhar Tata, Fabian Amman, Vinay Pawar, Michael T. Wolfinger, Siegfried Weiss, Susanne Häussler, Udo Bläsi

**Affiliations:** ^1^Department of Microbiology, Immunobiology and Genetics, Max F. Perutz Laboratories, University of Vienna, Vienna, Austria; ^2^Institute of Theoretical Chemistry, University of Vienna, Vienna, Austria; ^3^Institute of Immunology, Hannover Medical School, Hannover, Germany; ^4^Department of Molecular Bacteriology, Helmholtz Center for Infection Research, Braunschweig, Germany; ^5^Institute of Molecular Bacteriology, Twincore, Center for Experimental and Clinical Infection Research, Hannover, Germany

**Keywords:** *Pseudomonas aeruginosa*, anaerobiosis, small RNA, denitrification, nitrite

## Abstract

*Pseudomonas aeruginosa* is an opportunistic pathogen that can thrive by anaerobic respiration in the lungs of cystic fibrosis patients using nitrate as terminal electron acceptor. Here, we report the identification and characterization of the small RNA PaiI in the *P. aeruginosa* strain 14 (PA14). PaiI is *a*naerobically *i*nduced in the presence of nitrate and depends on the two-component system NarXL. Our studies revealed that PaiI is required for efficient denitrification affecting the conversion of nitrite to nitric oxide. In the absence of PaiI anaerobic growth was impaired on glucose, which can be reconciled with a decreased uptake of the carbon source under these conditions. The importance of PaiI for anaerobic growth is further underlined by the observation that a *paiI* deletion mutant was impaired in growth in murine tumors.

## Introduction

*Pseudomonas aeruginosa (Pae)* is an opportunistic human pathogen that causes severe infections in immunocompromised individuals, burn patients, and patients suffering from cystic fibrosis (CF). *Pae* can form biofilms in the lungs of CF patients under microaerobic or anaerobic conditions, underscoring the importance of anaerobic metabolism for pathogenicity (Yoon et al., 2002; Hassett et al., 2009; Kolpen et al., 2014). The switch of *Pae* from aerobic to anaerobic growth is controlled by the master regulator ANR (Zimmermann et al., 1991), which responds to oxygen limitation through its [4Fe-4S]^+2^ cluster (Ye et al., 1995). ANR acts as an upstream activator of the denitrification pathway by stimulating transcription of the nitrate-responsive two component regulatory system NarXL and the nitric oxide-dependent regulator DNR. In conjunction with NirQ, transcription of which is activated by DNR, these transcriptional regulators control a regulatory cascade, which results in the expression of genes encoding enzymes required for the reduction of nitrate to dinitrogen in four consecutive steps (Supplementary Figure S1; Schreiber et al., 2007).

During the last decade numerous small non-coding RNAs (sRNAs) have been identified in different pathogens, such as *Escherichia coli, Listeria monocytogenes, Staphylococcus aureus, Vibrio cholera*, and *Pae* (Gottesman and Storz, 2011; Caldelari et al., 2013). In general, bacterial sRNAs are induced by different environmental cues and their role in post-transcriptional regulation permits a fast adaptation to stressors and/or habitats (Hoe et al., 2013). The two major mechanisms by which sRNAs modulate gene expression includes base-pairing with target mRNAs or sequestration of a regulatory protein (Storz et al., 2011). At least in Enterobacteriaceae it is well-established that base-pairing sRNAs often require the RNA chaperone Hfq. Hfq fulfills several functions in post-transcriptional regulation. It can stabilize small regulatory RNAs (sRNAs) and facilitate annealing between sRNAs and their target mRNAs. The latter mode of action may result either in translational repression accompanied by degradation of both RNAs or in translational activation and consequently in stabilization of the mRNA (Vogel and Luisi, 2011).

Although many regulatory RNA candidates have been identified in different *Pae* strains (Livny et al., 2006; Sonnleitner and Haas, 2011; Ferrara et al., 2012; Wurtzel et al., 2012), only a few of them such as RsmW/RsmY/RsmZ, CrcZ, PhrS, NrsZ, and EsrA have been functionally characterized. The RNAs CrcZ (Sonnleitner and Bläsi, 2014) and RsmW/RsmY/RsmZ (Lapouge et al., 2008; Miller et al., 2016) function by sequestering regulatory proteins. On the other hand, the *bona fide* sRNAs PhrS (Sonnleitner et al., 2011), PrrF1-2 (Reinhart et al., 2015), NrsZ (Wenner et al., 2014), and EsrA (Ferrara et al., 2015) base-pair with and regulate their cognate target mRNAs. The *Pae* O1 sRNA PhrS, which has been implicated in the synthesis of the *Pseudomonas* quinolone signal (PQS), was hitherto the only identified sRNA whose expression depends on the anaerobic regulator ANR (Sonnleitner et al., 2011). In *E. coli*, the sRNA FnrS has been reported to be induced under hypoxic conditions in an FNR dependent manner. It was shown to regulate at least 32 mRNAs, many of which encode enzymes involved in central and energy metabolism, while a few encode enzymes required for aerobic respiration (Boysen et al., 2010; Durand and Storz, 2010). Hence, Durand and Storz (2010) suggested that FnrS serves to increase the efficiency of anaerobic metabolism by repressing functions that are not required under these conditions.

Given the importance of anaerobic biofilms in *Pae* pathogenicity, in this study we aimed at identifying novel regulatory sRNAs induced during anaerobiosis. We first re-analyzed a RNA_Seq_ based transcriptome study performed with the clinical isolate *P. aeruginosa* 14 (PA14) (Tata et al., 2016) for sRNA candidates that are induced at 30 min and 96 h after oxygen depletion, respectively. Among already known and new differentially abundant sRNA candidates this survey revealed the putative sRNA PA14_13970.1, which we termed PaiI. We show that PaiI transcription depends on the two component system NarXL. Further studies revealed that PaiI is required for optimal anaerobic growth on glucose and that it is required for a rapid conversion of NO_2_ to NO. A PA14 *paiI* deletion mutant showed an increased accumulation of nitrite and decreased nitrite reductase activity. Over-production of the transcriptional activator DNR rescued the *paiI* deletion phenotype. However, neither PaiI nor DNR over-production affected the levels of the nitrite reductase which suggests that PaiI indirectly impacts on denitrification. The importance of PaiI for anaerobic growth was further underlined by the observation that a *paiI* deletion mutant was impaired in growth in transplanted murine tumors that consist of hypoxic/anaerobic areas.

## Materials and methods

### Bacterial strains and growth conditions

Strains and plasmids used in this study are listed in Supplementary Table S1. Unless indicated otherwise, the *Pae* strains were grown at 37°C in basal salt medium (BSM) (Durham and Phibbs, 1982) supplemented with glucose or other carbon sources specified in the text (final concentration 20 mM). Anoxic growth was performed in an anaerobic chamber (95% N_2_, 5% H_2_). For growth under anoxic conditions the medium was supplemented with 100 mM KNO_3_. The *E. coli* strain DH5α was used for the construction of plasmids. When required, the following concentrations of antibiotics were used: 15 μg/ml gentamycin, 100 μg/ml ampicillin, and 25 μg/ml tetracycline for *E. coli*; 50 μg/ml gentamycin, 250 μg/ml carbenicillin and 100 μg/ml of tetracycline for *Pae*.

### Construction of PA14Δ*paiI* and PA14Δ*narL-paiI*

A markerless in-frame deletion of the *paiI* gene was constructed by homologous recombination (Ye et al., 1995). The PA14 genome coordinates referred to below are taken from http://www.pseudomonas.com/. First, the 717 bp upstream (PA14 genome coordinates: 1.198.245-1.198.961) and 700 bp downstream sequences (PA14 genome coordinates: 1.199.048-1.199.747) of the *paiI* gene were PCR-amplified using the primer pairs C99/E99 and D99/F99 (Supplementary Table S2), respectively. The PCR products were then linked by an overlap PCR to generate a fragment containing the in-frame deletion in *paiI*. The linked product was inserted into the KpnI and XbaI sites of plasmid pME3087 (Supplementary Table S1). The *paiI* deletion (PA14 genome coordinates: 1.198.962-1.199.047) was verified by DNA sequencing. The corresponding plasmid was then transformed into PA14 with the aid of *E. coli* strain HB101 (pRK2013). Single-crossover and double-crossover mutants were selected based on tetracycline resistance and sensitivity (Ye et al., 1995). The *paiI* gene deletion was confirmed by means of PCR.

Deletion of the NarL binding site within the *paiI* promoter sequence (PA14 genome coordinates: 1.199.107-1.199.115) was performed as described above. The primer pairs C99/P105 and D99/Q105 (Supplementary Table S2) were used for amplification of the upstream (1.198.245-1.199.106) and downstream (1.199.116-1.199.747) sequences of the NarL binding motif, respectively. The deletion of the NarL binding site within the *paiI* promoter in strain PA14Δ*narL-paiI* was verified by means of PCR and subsequent DNA sequencing.

### Construction of plasmids

DNA cloning and plasmid preparations were performed according to standard methods (Sambrook and Russell, 2001). The *paiI* expression plasmid pME-*paiI* was generated by PCR amplification of the *paiI* gene using PA14 genomic DNA as template and primers P107 and H99 (Supplementary Table S2). The PCR products were cleaved with SmaI and PstI and then ligated into the corresponding sites of plasmid pME4510-1 (Supplementary Table S1). Plasmid pME4510-1 was derived from plasmid pME4510 (Rist and Kertesz, 1998), wherein the *lacI*^*Q*^*-*P_*tac*_ region was PCR amplified from plasmid pMMBH67HE (Fürste et al., 1986) using the oligonucleotides Q107 and L85 (Supplementary Table S2). The resulting PCR products were then digested with SmaI and EcoRI and ligated into the corresponding sites of pME4510 to generate pME4510-1

The plasmids pMMB-*anr*, pMMB-*dnr*, and pMMB-*nirQ* were constructed by means of PCR amplification of the respective genes using PA14 genomic DNA as template and the primers listed in Supplementary Table S2. The PCR products were cleaved with HindIII and BamHI, and then ligated into the corresponding sites of vector pMMBΔ*rbs* (Sonnleitner and Bläsi, 2014). The forward primers used for PCR amplification of either gene comprised a ribosome binding site (see Supplementary Table S2).

### Northern-blot analysis

Total RNA was isolated from cultures using the TRIzol Reagent (Ambion) according to the manufacturer's instructions. For Northern-blot analysis, 10 μg of total RNA were heated at 65°C for 5 min in loading buffer (5 mM EDTA, 0.025% xylene cyanol, 0.025% bromophenol blue dissolved in formamide) and resolved on 8% polyacrylamide/8 M urea gels. The RNA was transferred onto Hybond N+ nylon membranes (GEHealthcare) using a semi-dry electroblotting apparatus (Transblot SD cell, BioRad) set at 13 V for 45 min followed by UV-cross-linking. A DNA oligonucleotide probe specific for PaiI (Supplementary Table S2) was 5′-end labeled with [γ-^32^P] ATP using T4 polynucleotide kinase. The [^32^P]-labeled oligonucleotide was heated at 95°C for 2 min, added to pre-hybridized membranes and incubated at 52°C overnight. Pre-hybridization and hybridization were performed in Roti® Hybrid Quick buffer (Carl Roth, Karlsruhe, Germany) supplemented with 0.1 μg/ml salmon sperm DNA. A 5S rRNA-specific oligonucleotide was used as a loading control, (Supplementary Table S2). The signals were visualized using a PhosphorImager (Molecular Dynamics).

### *In Vitro* transcription

For *in vitro* transcription of *paiI* (126 nt) and *paiI-1* (141 nt: used for sequencing reactions in primer extension analyses), the AmpliScribe T7-Flash Transcription Kit (Epicentre Biotechnologies) was used according to the manufacturer's instructions. PCR fragments used for *in vitro* transcription were generated with the primer pairs H109/I86 *paiI* (126 nt) and Q106/I86 *paiI-1* (141 nt) using PA14 genomic DNA as template (Supplementary Table S2). The forward primers contained a T7 promoter sequence.

### Primer extension analysis

The primer extension analysis was performed as described (Ausubel et al., 1997). Total RNA from B-96 cultures was prepared using the TRIzol Reagent (Ambion) according to the manufacturer's instructions. Primer R105 (Supplementary Table S2) was 5′-end labeled with [γ-^32^P] ATP using T4 polynucleotide kinase (Thermo Fisher Scientific) and annealed to 10 μg of total RNA. Primer extension reactions were carried out with avian myeloblastosis virus (AMV) reverse transcriptase (Promega). The extension products were loaded onto a 8 M urea−8% polyacrylamide gel next to the sequencing reactions performed with the same primer using *in-vitro* transcribed PaiI-1 (141 nt) sRNA as template.

### Enzymatic probing

Secondary structure probing of the sRNA PaiI was carried out as described (Franch et al., 1999). Briefly, 0.05 pmol of 5′ end-^32^P labeled *in vitro* transcribed PaiI RNA was denatured for 3 min at 85°C and slowly cooled to room temperature. Then, the RNA was incubated for 0, 10, and 15 min in reaction buffer (10 mM Tris, 60 mM NH_4_Cl, 6 mM β-mercaptoethanol, 2 mM magnesium acetate, pH 7.4) containing 0.2 U of RNase T1 (Sigma-Aldrich). The alkaline ladder was generated by incubation of 0.05 pmol 5′ end-labeled RNA at 85°C for 3 min in reaction buffer (10 mM Tris, 60 mM NH_4_Cl, 6 mM, β-mercaptoethanol, 2 mM Magnesium acetate, pH 7.4) containing 35 mM NaHCO_3_ (pH 9.0). The reactions were stopped by adding equal volumes of loading dye (5 mM EDTA, 0.025% xylene cyanol, 0.025% bromophenol blue) and analyzed on 8 M urea−8% polyacrylamide gels. The signals were visualized using a PhosphorImager (Molecular Dynamics).

### PaiI synthesis and stability

To monitor transcription of *paiI* after shift from aerobic to anaerobic conditions, PA14 was first grown aerobically in SCFM (Palmer et al., 2007) supplemented with 100 μM FeSO_4_ to an OD_600_ of 0.45. Then KNO_3_ was added to a final concentration of 100 mM, followed by incubation under anaerobic conditions. Total RNA was isolated at times 15, 30, 60, and 120 min to detect the PaiI levels by Northern-blotting as described above.

To measure the stability of PaiI after shift from anaerobic to aerobic conditions, PA14 was first grown in 50 ml flasks for 96 h under anaerobic conditions in SCFM in the presence of 100 mM KNO_3_ and 100 μM FeSO_4_ without shaking. Then, the cultivation was continued with shaking under aerobic conditions. Total RNA was isolated at times 15, 30, 60, and 120 min after the shift and PaiI was detected by Northern-blotting as described above.

### Determination of the CFU

Overnight cultures of PA14 were inoculated in fresh BSM medium supplemented with 20 mM glucose at an initial OD_600_ of 0.05. Cultures were grown aerobically to an OD_600_ of 0.45, at which, KNO_3_ (100 mM final concentration) was added. The cultures were then transferred to the anaerobic chamber. A sample of each culture was withdrawn before the shift (0 h) and after 2, 4, 6, and 8 h. The samples were serially diluted and plated on LB agar plates. The plates were incubated overnight aerobically at 37°C to determine the CFU. All experiments were performed in triplicate and the results are presented as mean ± standard deviation (SD).

### Glucose uptake

The glucose concentration present in the supernatant of the cultures was measured by using the glucose (GO) assay kit (Sigma-Aldrich: GAGO20-1KT). The cultures were grown under the same conditions as used for determination of the CFU. The cultures were then transferred to the anaerobic chamber. One milliliter sample of each culture was withdrawn before the shift (0 h) and 1 and 2 h thereafter. The samples were centrifuged at 4,000 rpm in a benchtop centrifuge for 10 min. The supernatant was filtered through a 0.2 μm filter and used for determination of the glucose concentrations. The amount of glucose present in the supernatant is inversely proportional to the amount of glucose taken up by the cultures. All experiments were performed in triplicates and the results are presented as mean ± standard deviation (SD).

### Determination of the nitrite concentration

The nitrite concentration in the supernatants of cultures was determined by the Griess assay (Nicholas and Nason, 1957). The cultures were grown under the same conditions as used for determination of the CFU. At different times, 1 ml of the cultures was centrifuged at 4,000 rpm in a benchtop centrifuge for 10 min. Equal amounts of 1% w/v sulfanilic acid dissolved in 20% HCl and 1.3 mg/ml naphthylenediamine dihydrochloride (NEDD) solutions were added to the supernatant. The reaction mixture was incubated for 5 min at room temperature to allow formation of the red azo dye. The absorbance was measured at 540 nm, and the nitrite concentration was determined by comparing the values to a standard curve obtained with different nitrite concentrations. All experiments were performed in triplicates and results are presented as mean ± standard deviation (SD).

### Determination of the nitrate reductase activity

The nitrate reductase activity was determined as described previously (Stewart and Parales, 1988). The cultures were grown under the same conditions as used for determination of the CFU. At the different time points, 1 ml samples were collected and centrifuged at 4000 rpm in a benchtop centrifuge for 10 min. The cell pellet was washed twice with 50 mM phosphate buffer, pH 7.2, and resuspended in 1 ml of the same buffer. The cells were permeabilized by addition of 50 μl toluene followed by shaking for 1 h at 400 rpm at 37°C. 800 μl of permeabilized cells were mixed with 100 μl of freshly prepared 0.5 mg/ml methyl viologen solution. Nitrate reduction was initiated by adding 100 μl of a solution containing 4 mg/ml sodium dithionite, 4 mg/ml sodium bicarbonate, and 100 mM KNO_3_. After 30 min of incubation, the reactions were stopped by vigorous shaking until the dark blue color disappeared. The amount of nitrite formed in the reaction mixture was measured by using the Griess assay (Nicholas and Nason, 1957). Enzyme activity is defined as the amount of nitrate reductase required to produce 1 nmol nitrite min^−1^ mg^−1^ protein. All experiments were performed in triplicates and the results are presented as mean ± standard deviation (SD).

### Determination of the nitrite reductase activity

The nitrite reductase activity was determined by measuring the reduction of KNO_2_ by methyl viologen (Moir et al., 1993). Sample preparation and processing was performed as specified above for the determination of the nitrate reductase activity. Nitrite reduction was initiated by adding 100 μl of a solution containing 4 mg/ml sodium dithionite, 4 mg/ml sodium bicarbonate and 200 μM KNO_2_. After 30 min, the assay was stopped by vigorous shaking until the dark blue color disappeared. The amount of nitrite remaining in the reaction mixture was measured by using the Griess assay (Nicholas and Nason, 1957), and was quantified by comparing the values to a standard curve obtained with nitrite. All experiments were performed in triplicates and repeated at least three times. All results are presented as mean ± standard deviation (SD).

### RNA_Seq_ library construction and sequence analysis

Total RNA was prepared from three biological replicates of PA14 and PA14Δ*paiI*, respectively. The overnight cultures were inoculated in fresh BSM medium supplemented with 20 mM glucose at an initial OD_600_ of 0.05. PA14 and PA14Δ*paiI* were first grown aerobically to an OD_600_ of 0.4, at which KNO_3_ (final concentration 100 mM) was added and then transferred to an anaerobic chamber. After 1 h of incubation, the cells were harvested for total RNA preparation using the TRIzol Reagent (Ambion) according to the manufacturer's instructions. Under these conditions anaerobiosis was ensured by employing an anaerobic indicator strip (Oxoid). The samples were DNase I treated, followed by phenol-chloroform-isoamyl alcohol extraction and ethanol precipitation. The Ribo-Zero™ rRNA Removal Kit (Illumina) was used to deplete rRNA from total RNA samples. Libraries were constructed using the NEBNext1Ultra™ Directional RNA Library Prep Kit from Illumina. 100 bp single end sequence reads were generated using the Illumina HiSeq 2000 platform at the Vienna Biocenter Core Facilities (http://www.csf.ac.at). The sequence reads were adaptor clipped and quality trimmed with trimmomatic (Bolger et al., 2014) with default parameters. Mapping of the samples against the UCBPP-PA14 reference genome (accession number NC_008463) was performed with Segemehl (Hoffmann et al., 2009, 2014). The uniquely mapped sequencing data were split by strand and further processed for automatic UCSC Genome Browser visualization with the ViennaNGS toolbox (Kent et al., 2002; Wolfinger et al., 2015). Read counting for subsequent DESeq2 (Love et al., 2014) differential gene expression analysis was performed with BEDtools (Quinlan and Hall, 2010). The raw sequencing reads were deposited in the European Nucleotide Archive as a study under the accession number PRJEB22593 (http://www.ebi.ac.uk/ena/data/view/PRJEB22593).

### Infection of tumor-bearing mice

The mouse experiments were performed as previously described (Pawar et al., 2015). Briefly, 7- to 8-week -old female BALB/c mice (Janvier, Germany) were injected intradermally with 5 × 10^5^ cells of the colon carcinoma cell line CT26. Mice bearing tumors of 150–200 mm3 diameter were infected intravenously (i.v.) with 5 × 10^6^ colony forming units (CFU) of the PA14 strains suspended in phosphate-buffered saline (PBS). All animal experiments were carried out with the permission of the lower Saxony authorities (LAVES—permission No. 33.9-42502-04-12/0713). At the indicated times, the mice were sacrificed and the tumors were homogenized in 2 ml of ice-cold 0.1% (v/v) Triton X-100/PBS using gentle MACS™ M-tubes and a dissociator from Miltenyi Biotec. The samples were serially diluted and plated on Luria-Bertani (LB) agar plates containing ampicillin (0.1 mg/ml).

### Western-blot analysis

The cultures were grown aerobically to an OD_600_ of 0.45 in minimal medium supplemented with 20 mM glucose followed by addition of KNO3 to a final concentration of 100 mM. The cultures were then transferred to the anaerobic chamber. One milliliter sample of each culture was withdrawn after 1 and 2 h. The samples were centrifuged at 4,000 rpm in a benchtop centrifuge for 10 min. Equal amounts of proteins were separated on a 12% SDS—polyacrylamide gel. The proteins were blotted onto a nitrocellulose membrane, which was blocked with 5% dried skimmed milk in TBS buffer. The membranes were probed with mouse anti-NirS (Nicke et al., 2013) and rabbit anti-S2 (laboratory stock) antibodies. The antibody-antigen complexes were visualized with alkaline-phosphatase conjugated secondary antibodies (Sigma) using the chromogenic substrates nitro blue tetrazolium chloride (NBT) and 5-Bromo-4-chloro-3-indolyl phosphate (BCIP).

## Results

### Identification of sRNA candidates in anoxic biofilms of PA14

**Figure 1:**
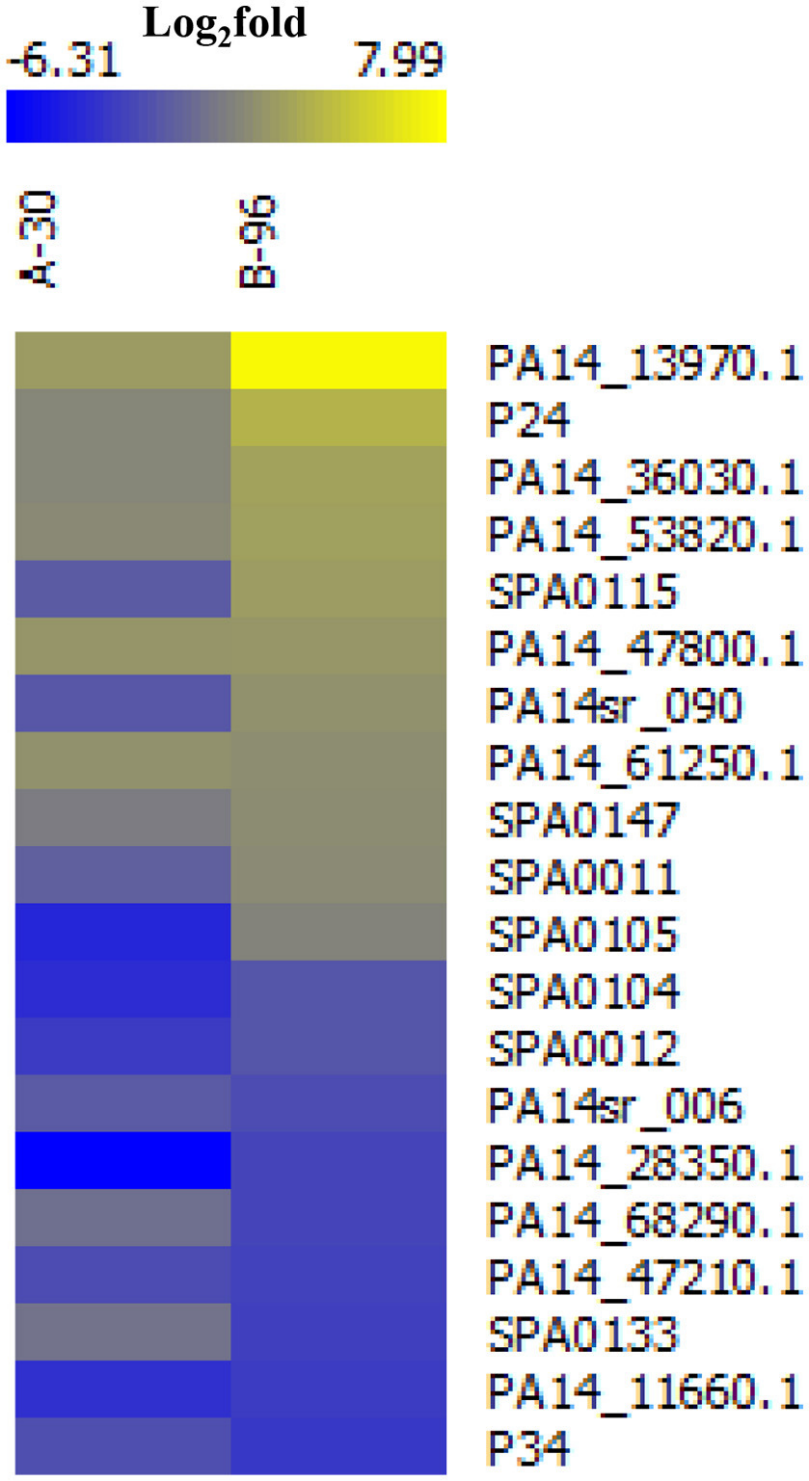
Heat map of differentially abundant novel/annotated sRNAs under the conditions A-30 and B-96 when compared with condition P. The color code shown in the scale at the top denotes log2-fold changes. Blue and yellow indicate a decrease and an increase in the sRNA levels, respectively.

With the aim to identify novel sRNAs that are up-regulated in anoxic biofilms we revisited a recently published RNA_Seq_-based transcriptome analysis, wherein the transcriptomes of PA14 grown under planktonic (P), anoxic conditions for 30 min (A-30) and anoxic biofilm conditions for 96 h (B-96) were compared (Tata et al., 2016). Based on the criteria that sRNA genes should preferably reside in the intergenic regions and/or in the 5′ or the 3′ UTR of annotated genes, we identified 20 sRNA candidates that are differentially abundant in A-30/B-96 conditions when compared with planktonic conditions. Of these, 11 had already been noticed previously and 9 represented novel sRNA candidates (Figure 1, Supplementary Table S3). Next, the Virtual Footprint tool of the PRODORIC database (http://www.prodoric.de) was used to identify possible ANR and NarL binding motifs in the promoter regions of the up-regulated sRNAs candidates. Among these sRNA candidates, SPA0115 contains a putative ANR binding site, whereas PA14_13970.1, PA14_36030.1, PA14_47800.1, SPA0147, and SPA0011 contain putative NarL signatures (Supplementary Table S3). Moreover, a co-immunoprecipitation experiment with Hfq-specific antibodies (Pusic et al., 2016) indicated that 7 of these 20 sRNA candidates might interact with Hfq (Supplementary Table S3).

### Detection and characterization of the anaerobically induced sRNA PaiI

Among all putative sRNAs, the sRNA candidate PA14_13970.1 located in the intergenic region between PA14_13970 and PA14_13990 (http://www.pseudomonas.com/) was most abundant in A-30 and B-96 conditions when compared with condition P (Figure 1, Supplementary Figure S2A). The putative sRNA was found to be highly conserved among *P. aeruginosa* strains (Zhang et al., 2000), and was termed PaiI (*P*seudomonas *a*naerobically *i*nduced RNA *I*).

**Figure 2:**
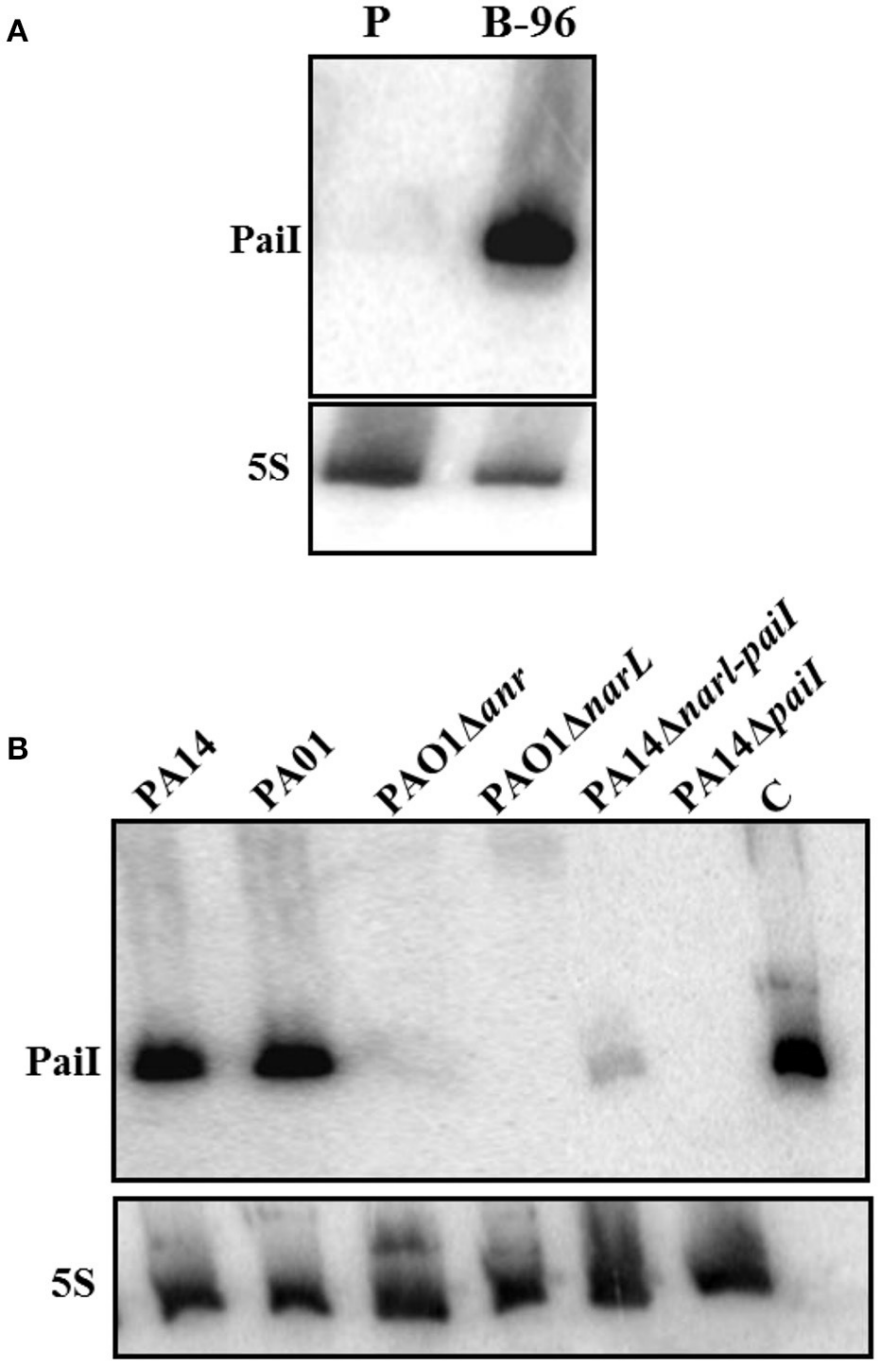
Anaerobic transcription of PaiI requires NarXL. **(A)** Expression of PaiI in PA14 B-96 cells. Total RNA was extracted from planktonically growing cells (P) and from anoxic biofilms (B-96) under the same conditions as described (Tata et al., 2016) and the PaiI was detected as described in Materials and Methods. **(B)** The NarXL two-component regulatory system is required for expression of PaiI. Detection of PaiI by Northern-blotting in total RNA extracted from PAO1 and PA14, and different mutants thereof grown in anoxic biofilms (B-96). 0.6 ng of *in vitro* transcribed PaiI (126 nt) was loaded as a control (C).

To verify the expression of PaiI under anaerobic conditions total RNA was isolated from PA14 P-cells and B-96 cells (Tata et al., 2016), and examined for the presence of PaiI by Northern-blotting. As anticipated PaiI was exclusively detected under anaerobic conditions in B-96 cells (Figure 2A).

Next, the 5′ end of PaiI was mapped by primer extension using total RNA prepared from PA14 B-96 cultures (Supplementary Figure S2B). This analysis indicated that PaiI transcription initiates with the A at nucleotide position 1.199.053 (Supplementary Figure S2C). The transcriptional start site is preceded by a σ^70^ recognition motif. Inspection of the secondary structure by RNAfold (rna.tbi.univie.ac.at/cgi-bin/RNAWebSuite/RNAfold.cgi) predicted a 3′-terminal stem-loop structure followed by a consecutive stretch of five U residues, indicative for a rho-independent terminator (Farnham and Platt, 1981). With this information, the length of PaiI was calculated with 126 nt, which complied with the size judged from Northern-blot experiments (not shown). No extensive secondary structures in addition to the predicted rho-independent terminator (Supplementary Figure S3A) was revealed by employing the RNA-fold algorithm (rna.tbi.univie.ac.at/cgi-bin/RNAWebSuite/RNAfold.cgi). This finding was supported by *in vitro* enzymatic probing using RNase T1, which cleaves after G residues. Although RNase T1 cleavage was not obvious at several positions, cleavage occurred at the majority of G residues preceding the terminal stem-loop structure (Supplementary Figure S3B).

### Transcription of PaiI is NarL dependent

The primary sequence of PaiI is highly conserved in all sequenced *Pae* strains (98–100% identity; Zhang et al., 2000). In addition, a NarL binding motif (Supplementary Figure S2C) was discerned upstream of a σ^70^ promoter for either *paiI* homolog. Therefore, we first tested the PaiI abundance in B-96 cells of *P. aeruginosa* O1 and in the isogenic mutant strains PAO1Δ*anr* and PAO1Δ*narL*, respectively. As judged by Northern-blot analyses (Figure 2B) PaiI was absent in the mutant strains, strongly suggesting that transcription is NarL dependent. To verify the NarL dependent transcription of PaiI in PA14, the NarL binding motif within the *paiI* promoter region (Supplementary Figure S2C) was deleted in strain PA14Δ*narL-paiI*. When compared with PA14, only minute amounts of PaiI were present in B-96 cells of PA14Δ*narL-paiI* (Figure 2B). In addition, PaiI was not detected in B-96 cells when nitrate was omitted from the medium (Supplementary Figure S4). Taken together, these experiments verified that *paiI* transcription requires the nitrate-responsive two component regulatory system NarXL, transcription of which in turn depends on the anaerobic regulator ANR (Schreiber et al., 2007).

### Synthesis and turnover of PaiI

**Figure 3:**
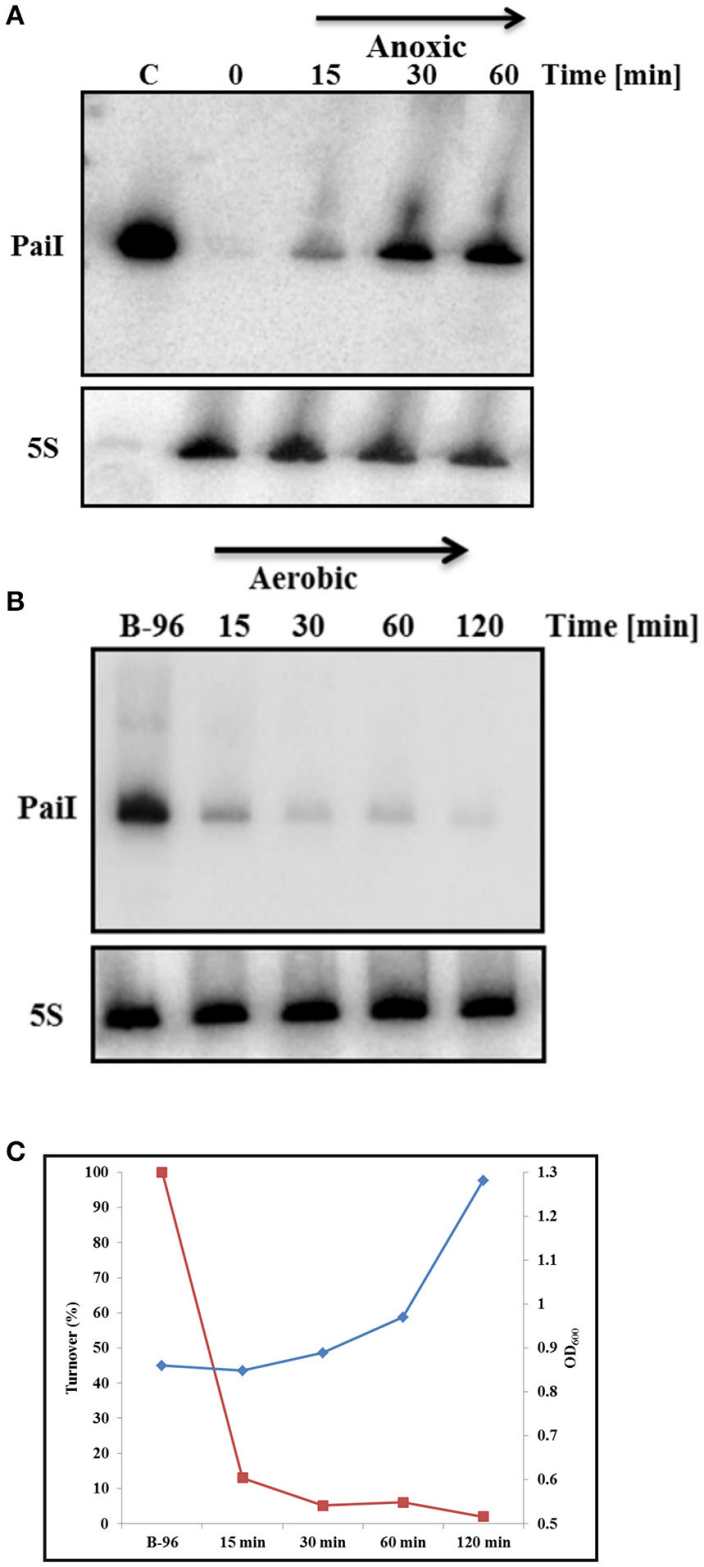
Synthesis and turnover of PaiI. **(A)** PA14 cultures were grown planktonically to an OD_600_ of 0.45 (0 min). Then, KNO_3_ was added to a final concentration of 100 mM and the cultures were shifted to anoxic conditions. Total RNA was extracted at the indicated times followed by the detection of PaiI by Northern-blotting. 0.6 ng of *in vitro* transcribed PaiI (126 nt) was loaded as a positive control (C). **(B)** PA14 B-96 cultures were shifted to aerobic conditions. Total RNA was extracted at the indicated times followed by the detection of PaiI by Northern-blotting. **(C)** Graphical presentation of PA14 growth (blue graph) and PaiI decay (red graph) based on the results shown in **(B)**.

Next, we studied the time course of induction of *paiI* after shift from aerobic to anaerobic conditions as described in Materials and Methods. Samples for isolation of total RNA were withdrawn after 15, 30, and 60 min. As shown in Figure 3A, PaiI was detectable after 15 min and was highly expressed 30 min after the shift to anaerobiosis. Next, the reverse experiment was performed. B-96 cells were shifted to aerobic conditions with vigorous shaking. As shown in Figures 3B,C, the PaiI levels declined after shift to aerobic conditions. As ANR and NarL are non-functional in the presence of oxygen and in the absence of KNO_3_, respectively (Schreiber et al., 2007), this observation indicated that PaiI is removed when its synthesis discontinues upon shift to aerobiosis.

### PaiI is required for anoxic survival of *Pae* when growing on glucose

**Figure 4:**
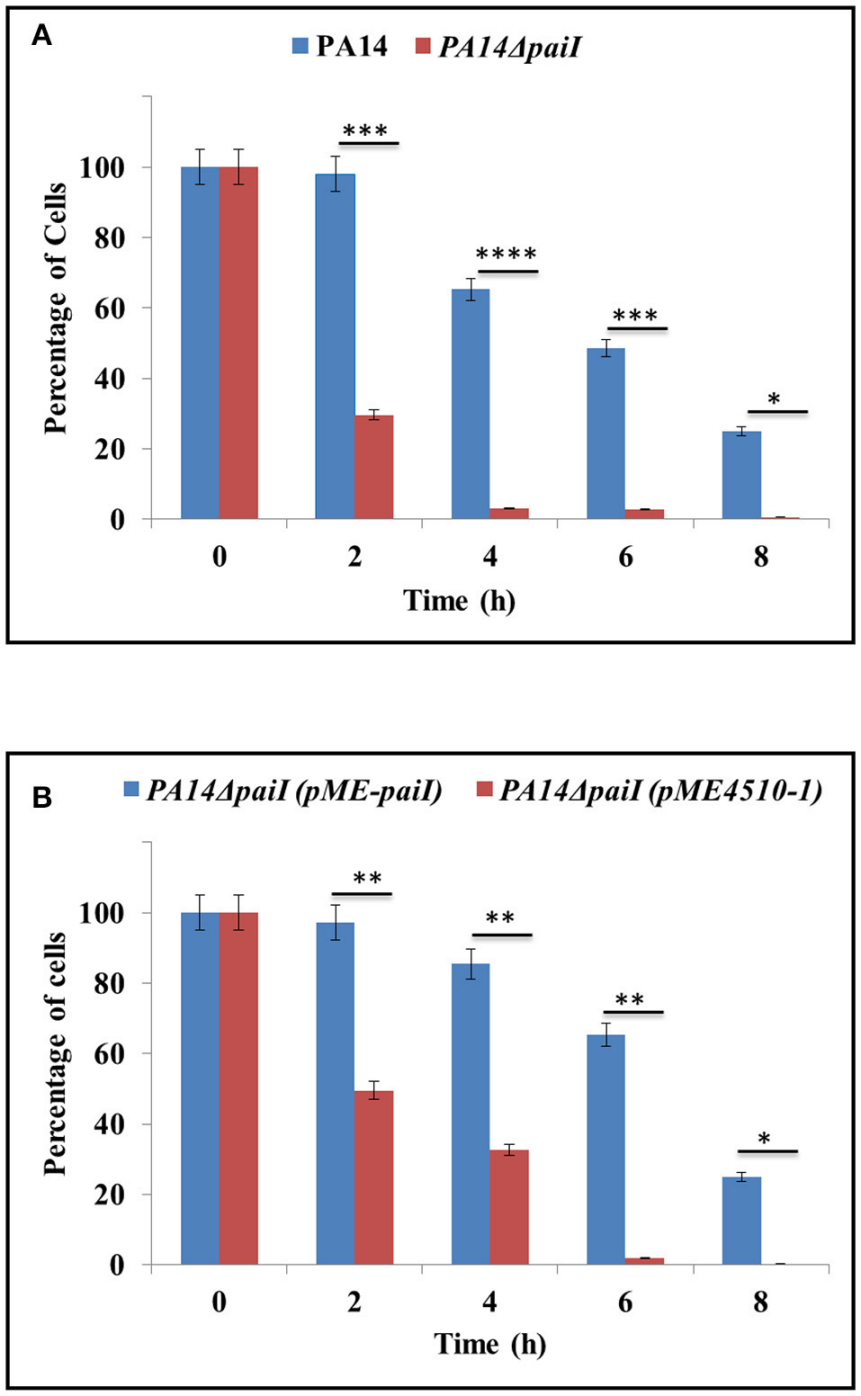
Anaerobic growth of PA14Δ*paiI*. The strains were grown in BSM medium supplemented with 20 mM glucose. The CFU at time 0 (shift from aerobic to anaerobic conditions and concomitant addition of KNO_3_ to a final concentration of 100 mM) was set to 100%. The bars represent the percentage in CFU at either time relative to time 0. The CFU were obtained by plating serial dilutions of the cultures at the indicated times on LB plates. **(A)** Decline in the CFU of PA14 and PA14Δ*paiI* after shift to anaerobiosis. **(B)** Decline in the CFU of PA14Δ*paiI* (pME-*paiI*) and PA14 (pME4510-1) after shift to anaerobiosis. At time 0, IPTG was added to a final concentration of 2 mM to both cultures to induce transcription of the plasmid borne *paiI* gene in strain PA14Δ*paiI*(pME-*paiI*). Depicted is mean ± SD, *n* = 3. Statistical analysis was performed using the unpaired student *t*-test (www.graphpad.com). ^****^p < 0.0001, ^***^p < 0.001, ^**^p < 0.01, ^*^p < 0.05.

As PaiI is only present during anoxic growth, we next asked whether it impacts on growth and viability under these conditions. To address this, a PA14 *paiI* deletion mutant was constructed, and growth of strain PA14Δ*paiI* in BSM medium supplemented with different carbon sources was compared with that of the wild-type strain PA14. These initial growth experiments revealed that anaerobic growth of PA14Δ*paiI* was only retarded after 96 h when glucose was provided as the sole carbon source (data not shown). To study this further, we determined the CFU of PA14 and PA14Δ*paiI* during growth in BSM medium supplemented with glucose and KNO_3_ at several times after shift to anaerobiosis. As shown in Figure 4A, PA14Δ*paiI* showed a reduced viability under anoxic conditions when compared to PA14. Ectopic expression of *paiI* in strain PA14Δ*paiI*(pME-*paiI*) rescued the phenotype and restored the CFU comparable to that of PA14 (Figure 4B).

### Uptake of glucose in PA14Δ*paiI*

**Figure 5:**
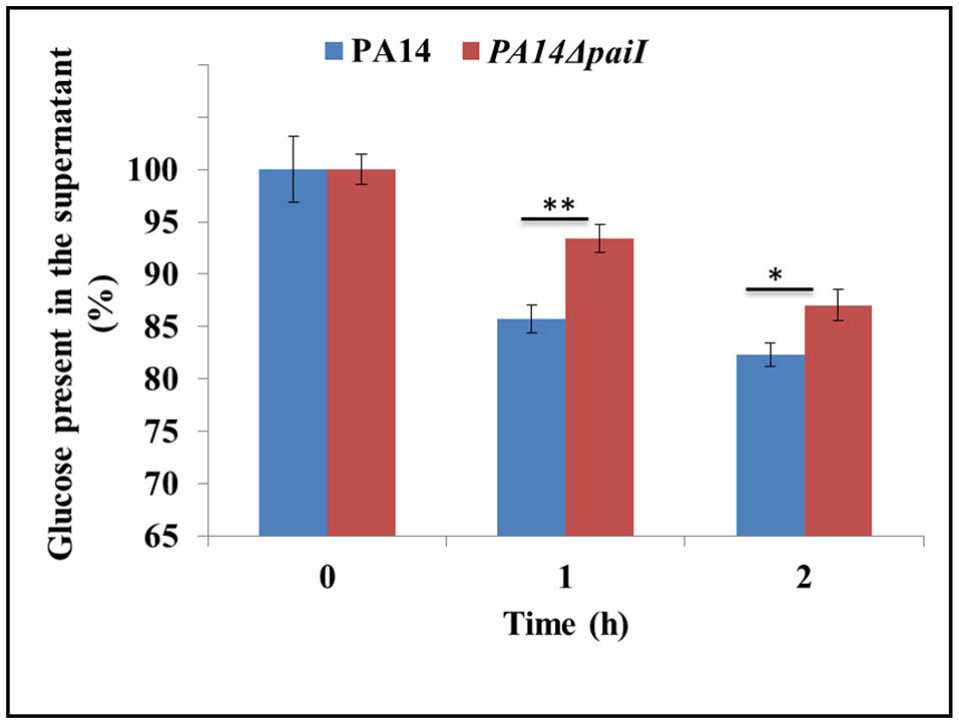
Reduced glucose uptake by strain PA14Δ*paiI*. The amount of glucose present in the supernatant of PA14 and PA14Δ*paiI* cultures was determined under the same conditions as described in the legend to Figure 4. The glucose concentration at time 0 (shift from aerobic to anaerobic conditions and addition of KNO_3_ to a final concentration of 100 mM) was set to 100%. The bars represent the percentage of glucose present at either time relative to time 0.). Depicted is mean ± SD, *n* = 9. Statistical analysis was performed using the unpaired student *t*-test (www.graphpad.com). ^**^p < 0.01, ^*^p < 0.05.

Since PA14Δ*paiI* showed reduced viability in glucose minimal medium, we next asked whether the observed phenotype can be attributed to a decreased glucose uptake. To test this, the amount of glucose present in the supernatants of PA14 and PA14Δ*paiI* was determined before (0 h) and 1 and 2 h after shift to anaerobiosis. As shown in Figure 5, the amount of glucose present in the supernatant of PA14Δ*paiI* was somewhat higher than that observed for PA14. These results implied that the sRNA might impact on glucose uptake under anaerobic conditions.

### Reduced nitrite reductase activity in PA14Δ*paiI*

As expression of *paiI* was nitrate dependent (Supplementary Figure S4), we tested whether PaiI impacts on the denitrification pathway. First, we asked whether PaiI affects the first step in denitrification, i.e., the conversion of NO_3_ to NO_2_, which is performed by the cytoplasmic nitrate reductase (Supplementary Figure S1). However, no significant difference in the nitrate reductase activity was observed in strains PA14 and PA14Δ*paiI* after shift to anaerobiosis (Supplementary Figure S5).

**Figure 6:**
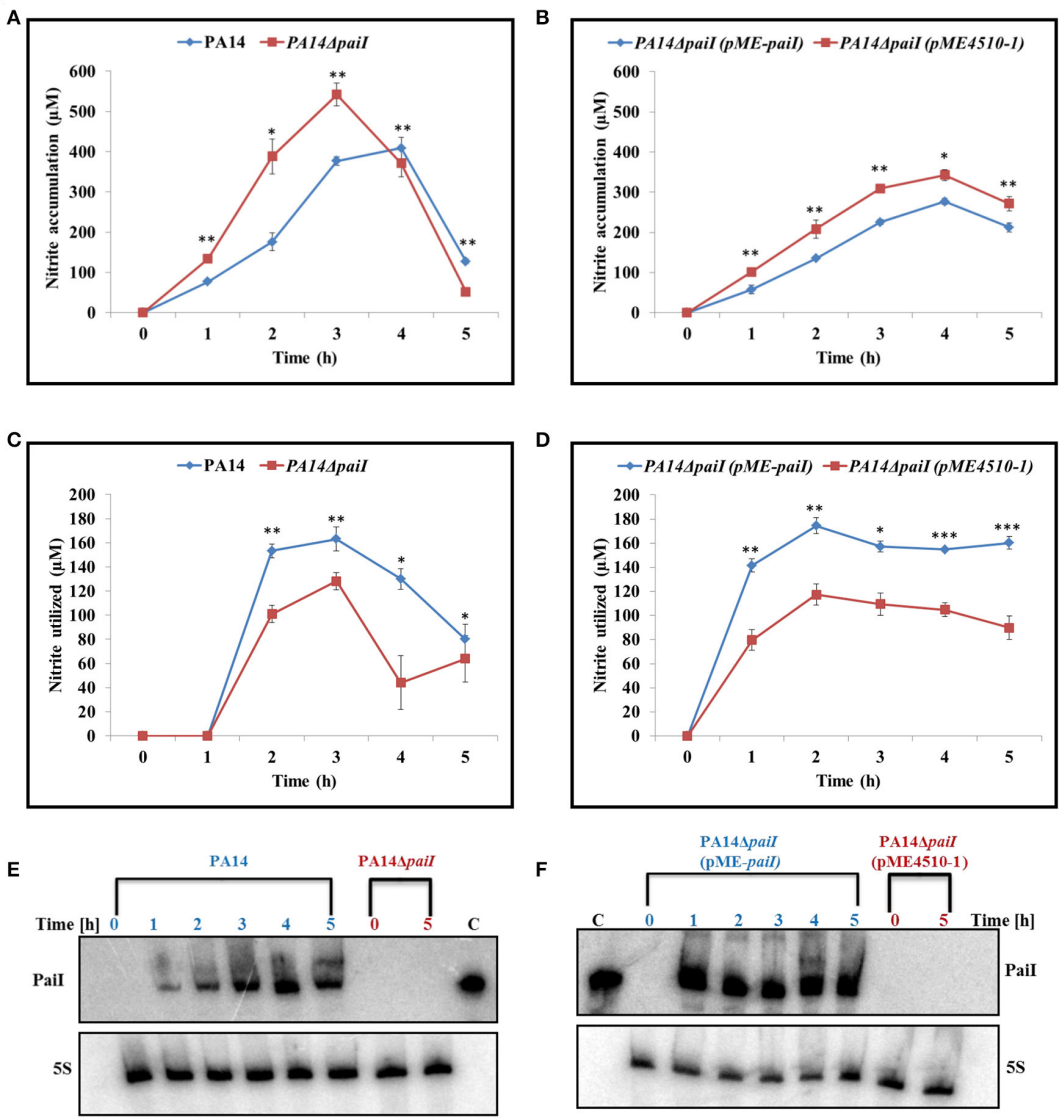
NO_2_ accumulation and nitrite reductase activity obtained with strains PA14, PA14Δ*paiI*, and PA14Δ*paiI*(pME-*paiI*). To determine the NO_2_ concentration in the supernatants **(A,B)** and the NO_2_ reductase activity **(C,D)**, the cultures were grown under the same conditions as described in the legend to Figure 4. Depicted is mean ± SD, *n* = 3. Statistical analysis was performed using the unpaired student *t*-test (www.graphpad.com). ^***^p < 0.001, ^**^p < 0.01, ^*^p < 0.05. **(E,F)** PaiI levels detected by Northern-blot analyses in total RNA isolated from the samples collected at the indicated times. For strains PA14Δ*paiI* and PA14Δ*paiI* (pME4510-1) only total RNA isolated at time 0 and 5 h were loaded onto the gel. 5S RNA levels determined by Northern-blotting served as a loading control.

Second, the NO_2_ accumulation was compared in the supernatants of PA14 and PA14Δ*paiI* at different times after shift to anaerobiosis. Up to 3 h after shift to anaerobiosis, there was significantly more NO_2_ accumulation observed with PA14Δ*paiI* than with the wild-type strain (Figure 6A). Ectopic expression of PaiI in strain PA14Δ*paiI*(pME-*paiI)* complemented the *paiI* deletion phenotype (Figure 6B). As the difference in the NO_2_ levels observed with PA14 and PA14Δ*paiI* were unlikely to result from a differential nitrate reductase activity (Supplementary Figure S5), we next tested whether PA14Δ*paiI* displays differences in the activity of the nitrite reductase, which converts NO_2_ to NO (Supplementary Figure S1). These enzymatic assays revealed a reduced nitrite reductase activity in PA14Δ*paiI* when compared with PA14 during the first 3 h after shift to anaerobiosis (Figure 6C). Ectopic expression of *paiI* in strain PA14Δ*paiI*(pME-*paiI)* again complemented this phenotype (Figure 6D).

### Overexpression of *dnr* alleviates the *paiI* deletion phenotype

**Figure 7:**
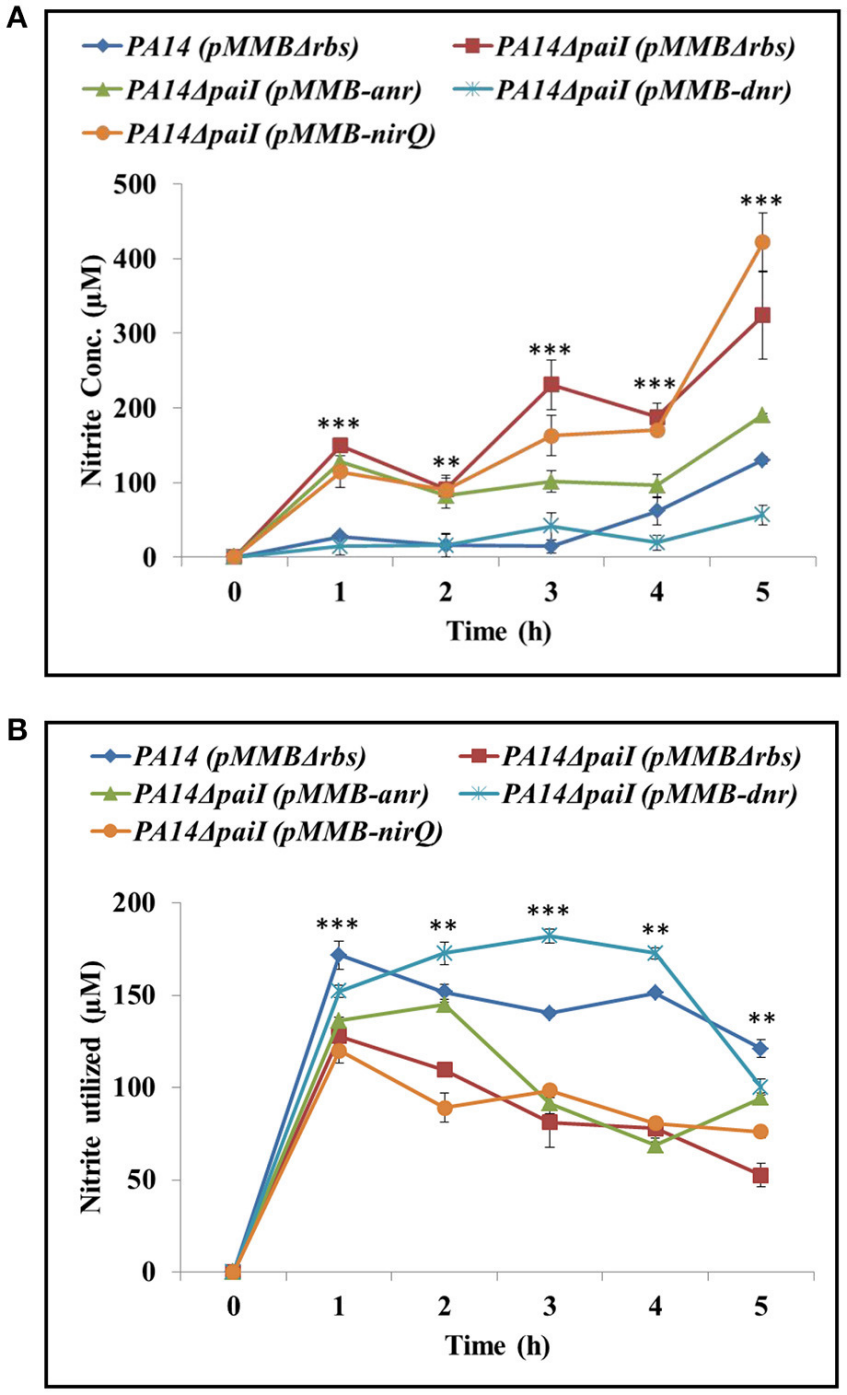
Ectopic expression of *anr* and *dnr* complement the Δ*paiI* phenotype. **(A)**, NO_2_ levels in the supernatants and **(B)** nitrite reductase activity determined with strains PA14Δ*paiI*(pMMB-*anr*), PA14Δ*paiI*(pMMB-*dnr*) and PA14Δ*paiI*(pMMB-*nirQ*). PA14 and PA14Δ*paiI* strains carrying the empty vector pMMBΔ*rbs* were used as a control. The cultures were grown under the same conditions as described in the legend to Figure 4. Depicted is mean ± SD, *n* = 3. Statistical analysis was performed using ordinary one-way ANOVA (www.graphpad.com). ^***^p < 0.001, ^**^p < 0.01.

As the PA14Δ*paiI* mutant showed a reduced nitrite reductase activity and higher levels of NO_2_ in the culture supernatant, we next asked whether increased synthesis of the transcriptional regulators ANR, DNR and NirQ can alleviate the *paiI* deletion phenotype. ANR positively regulates *dnr*, whereas DNR and NirQ are direct positive regulators of the *nir* operon, encoding the subunits of the nitrite reductase (Supplementary Figure S1; Schreiber et al., 2007). The corresponding genes were mounted into the expression vector pMMB67HE under transcriptional control of the *tac* promoter. The nitrite accumulation and nitrite reductase activity were then compared in strains PA14Δ*paiI*(pMMBH67E), PA14Δ*paiI*(pMMB-*anr*), PA14Δ*paiI*(pMMB-*dnr*) and PA14Δ*paiI*(pMMB-*nirQ)*. As shown in Figures 7A,B, over-expression of *dnr* restored the nitrite reductase activity in PA14Δ*paiI*(pMMB-*dnr*) to wild-type levels, which was accompanied by a reduced NO_2_ accumulation. A similar effect was observed after *anr* over-expression albeit to a lower extend. In contrast, over-expression of *nirQ* did not impact on the NO_2_ accumulation and nitrite reductase activity (Figures 7A,B).

### Comparative transcriptome analysis of PA14 vs. PA14Δ*paiI*

In order to identify target gene(s) for PaiI, an RNA_seq_ based transcriptome analysis was performed with total RNA isolated from PA14 and PA14Δ*paiI* after anaerobic growth for 1 h in BSM medium supplemented with glucose and KNO_3_. Only 19 transcripts (15 up- and 4 down-regulated) showed a ≥± 1.5-fold change in PA14 when compared with PA14Δ*paiI* (Supplementary Table S4). However, none of the encoded genes are directly involved in the denitrification pathway. The only transcripts that might indirectly affect the denitrification pathway are those involved in sulfur metabolism (*cysA, cysI*, and *cysN*), as iron sulfur clusters are required for the function of ANR and of the nitrate reductase activity (Zumft, 1997; Yoon et al., 2007). However, as the nitrate reductase activity was not impaired in the PA14Δ*paiI* strain (Supplementary Figure S5), we did not follow up the idea that a scarcity of sulfur is responsible for the Δ*paiI* phenotype. In addition, two chaperone genes (*ibpA* and *groES*) were significantly more abundant in PA14 when compared to PA14Δ*paiI*. IbpA is a heat shock protein, which is induced in response to several stress conditions like oxygen limitation in *Bacillus subtilis* (Hecker et al., 1996), toluene and other stresses in *Pseudomonas putida* (Matuszewska et al., 2008). We next investigated whether IbpA might impact on denitrification by comparing the anaerobic growth of PA14 with that of a PA14Δ*ibpA* mutant strain in BSM medium supplemented with glucose. Growth was not impaired in the deletion strain (not shown), which apparently eliminates the possibility that this chaperone is involved in denitrification. Moreover, we observed that the *mexG* gene, which appears to be indirectly regulated by DNR (Trunk et al., 2010) was also down-regulated in PA14Δ*paiI*. In addition, the *rsmA* gene, encoding the translational regulator RsmA that is involved in regulation of virulence genes (Brencic and Lory, 2009) was up-regulated. Of the 4 transcripts that were down-regulated in the presence of PaiI, two encode hypothetical proteins (PA14_11670 and PA14_13960). The other two genes encode a putative transposase (PA14_13970) and aquaporin Z porin (*aqpZ)*, respectively. Aquaporin Z is selectively permeable to water and plays a role in osmoregulation (Calamita, 2000), however, it has so far not been linked to the denitrification pathway. In contrast to *E. coli* FnrS, which was reported to repress functions that are not required during anaerobiosis (Durand and Storz, 2010), we did not observe a significant down-regulation of such functions.

### PA14Δ*paiI* growth is impaired in murine tumors

**Figure 8:**
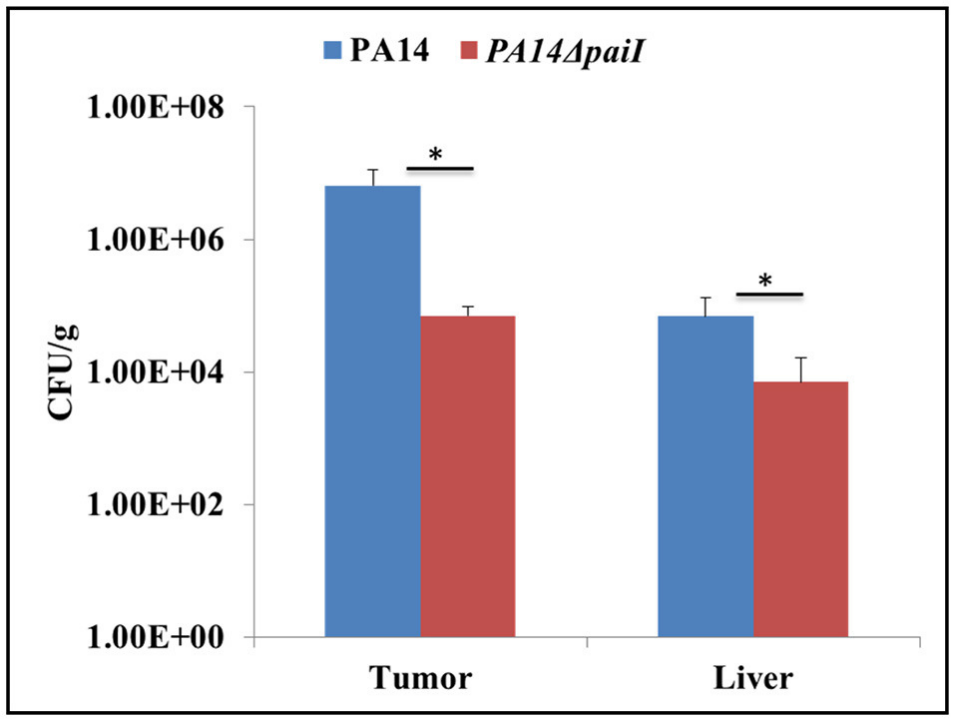
Bacterial colonization of CT26 tumors. Tumor-bearing mice were infected intravenously with *P. aeruginosa* PA14 and PA14Δ*paiI*. At 24 h the tumor and liver were homogenized and the CFU per gram of tissue were determined. Depicted is mean ± SD, *n* = 5. Statistical analysis was performed using the unpaired student *t*-test (www.graphpad.com). ^*^p < 0.05.

The above findings suggested that PaiI positively impacts on the denitrification pathway. This prompted us to test whether the *paiI* deletion strain is impaired in growth in CT26 murine tumors. In this model the capability to survive in hypoxic/anaerobic conditions within the tumor is crucial for tumor colonization (Komor et al., 2012). The BALB/c mice bearing an ectopic CT26 tumor were intravenously (i.v) infected with either PA14 or PA14Δ*paiI*. At 24 h post-infection (p.i), bacterial colonization of the tumor was assessed by determination of the CFU. As shown in Figure 8, the *paiI* deletion strain was impaired in colonizing the tumor. Thus, this experiment again underlined the function of PaiI in adaptation to anaerobic denitrification conditions.

## Discussion

Here, we report the first study on a *Pseudomonas* sRNA that is induced during nitrate respiration in a NO_3_-/NarXL dependent manner (Figure 2, Supplementary Figure S4). In strain PA14, the *paiI* gene is situated in the intergenic region between PA14_13970 and PA14_13990 (Supplementary Figure S2A), which encodes a putative transposase and a component of a putative ABC transporter, respectively (Zhang et al., 2000). While the synteny of *paiI* and PA14_13990 is highly conserved in all sequenced 138 *P. aeruginosa* strains (Zhang et al., 2000), the putative transposon gene is only present in 24 (Zhang et al., 2000). PaiI was detectable after shift to anaerobiosis (Figure 3A) and was rapidly degraded after shift to aerobic conditions (Figure 3B). Unfortunately, the use of mutant strains of the Liberati transposon library (Liberati et al., 2006) with insertions in the genes encoding the enzymes RNase E, CafA, RNase III, RNase R, and RNase HII did not allow to pin down a particular RNase involved in degradation of the sRNA, as it was rapidly degraded upon aerobic shift in either mutant (not shown).

A detectable phenotype of the *paiI* deletion strain was only observed during anaerobiosis when glucose was provided as the sole carbon source (Figure 4), which can be reconciled with a somewhat reduced uptake of glucose (Figure 5). However, the transcriptome analysis did not reveal significant differences in the transcript levels of the major functions involved in glucose uptake such as *oprB, gltB,F,G*,*K* in PA14 and PA14Δ*paiI* (Supplementary Table S4), which indicated that PaiI does not directly impact on the expression of these genes. In the absence of PaiI, an increased transient accumulation of nitrite was observed in the medium, which concurred with a reduced activity of the nitrite reductase (Figure 6). It has been reported that glucose uptake under denitrifying conditions corresponds to the maximal levels of nitrate and nitrite reductase activities, and coincided with the disappearance of nitrite from the medium (Williams et al., 1978). The latter authors interpreted this as showing that glucose uptake is directly linked to the activity of these enzymes, i.e., to the levels of nitrate and nitrite. As the activity of the nitrate reductase was not impaired in PA14Δ*paiI* (Supplementary Figure S5), it seems likely that the lowered uptake of glucose resulted from the reduced activity of the nitrite reductase, i.e., from increased levels of nitrite. However, the molecular details underlying nitrite-linked glucose transport remain unknown in *Pae*.

The RNA_Seq_ based transcriptome analysis did not reveal significant differences in the levels of the *nir* transcripts in PA14Δ*paiI* when compared with the wild-type strain (Supplementary Table S4). However, the transcript levels may not always mirror the translational output of a given gene. Among other regulatory events, sRNAs can positively regulate translation by interfering with intramolecular secondary structures that obstructs the translational initiation signals, i.e. the Shine and Dalgarno (SD) sequence or the start codon (Reichenbach et al., 2008; Sonnleitner et al., 2011). As indicated in Supplementary Figure S6A, PaiI displays sequence complementarity to a region upstream of the SD sequence of *nirS* mRNA that might form a stem-loop structure, which could potentially impact on its translational efficiency. To test whether PaiI positively affects translation of *nirS*, the nitrite reductase levels were assessed by quantitative western-blotting with anti-NirS antibodies. No apparent difference in the NirS levels were observed in strain PA14 when compared with strain PA14Δ*paiI* (Supplementary Figure S6B), excluding the possibility that PaiI directly regulates the translational output of *nirS* mRNA.

The increased nitrite levels in the medium and the reduced nitrite reductase activity observed with the PA14Δ*paiI* strain were alleviated by ectopic expression of the *anr* and *dnr* genes (Figure 7). Hence, the observed restoration of the nitrite reductase activity could be simply based on a gene dosage effect, i.e., an increased transcription of *nirS* mRNA through over-production of DNR (see Supplementary Figure S1) followed by a concomitant increase of the NirS levels. However, we did not observe an increased production of NirS in strain PA14Δ*paiI* harboring plasmid pMMB-*dnr* (Supplementary Figure S6B). Interactome studies revealed that a number of proteins involved in several metabolic pathways associate together with denitrification enzymes to form a nitrate respirasome (Borrero-De Acuña et al., 2016). In addition, there are a number of accessory proteins that have been implicated in denitrification by genetic analyses (Zumft, 1997) and fitness studies (Vaccaro et al., 2015). This raises the possibility that both, DNR and PaiI, regulate a hitherto unknown accessory factor that affects the function of nitrite reductase. Ectopic expression of *anr*, encoding the anerobic regulator ANR, resulted also in partial complementation of the Δ*paiI* phenotype (Figure 7). The simplest explanation is the positive control of DNR by ANR (see Supplementary Figure S1; Schreiber et al., 2007) as all genes that are DNR dependent are also dependent on ANR (Trunk et al., 2010). In contrast, ectopic expression of *nirQ* did not restore the Δ*paiI* phenotype albeit it was shown to positively control *nirS* transcription (Hayashi et al., 1998). We can only speculate that NirQ does not impact on production of the hypothetical accessory factor for NirS function.

There are other possibilities how PaiI might indirectly affect the activity of nitrite reductase. One possibility for the reduced nitrite reductase activity observed in PA14Δ*paiI* are the increased nitrite levels, which might negatively impact on the heme moiety of the enzyme (Rowe et al., 1977). Because of its toxicity nitrite is excreted to the periplasm, where the nitrite reductase converts it to nitric oxide (Schreiber et al., 2007). Thus, the compartmentalization of nitrite might explain why the activity of the nitrite reductase was affected in the PA14Δ*paiI* but not that of the inner membrane bound nitrate reductase, the NarI subunit of which also contains a heme group (Van Alst et al., 2009). Moreover, Yoon et al. (2002) have reported that a *Pae* O1*oprF* mutant lacked nitrite reductase activity, whereas the underlying mechanism remains speculative. However, the transcriptome data did not reveal significant changes in the transcript levels of *oprF* and no apparent differences in the OprF levels were observed by quantitative Western-blotting (not shown) in the absence or presence of PaiI, respectively. Thus, these data would argue against the possibility that PaiI acts on the nitrate reductase activity through regulation of *oprF*.

Although under laboratory conditions the Δ*paiI* phenotype was only obvious when glucose was used as the sole carbon source, it was striking that the absence of PaiI resulted in a fitness burden in the mouse tumor model (Figure 8). A PA14 *anr* mutant was found to colonize the tumor at lower bacterial numbers and did not colonize the hypoxic/anaerobic central necrotic areas of the tumor which emphasized the importance to adapt to microaerophilic/anaerobic growth conditions for efficient tumor colonization (Komor et al., 2012). Comparative transcriptional profiling of *Pae* indicated physiological similarity of the bacteria in the murine tumor model and in the cystic fibrosis (CF) lung (Bielecki et al., 2013). Thus, it appears possible that PaiI also contributes to the adaptation of *Pae* to conditions present in the CF lung, where *Pae* can grow anaerobically (Kolpen et al., 2014).

## Author contributions

Conceived and designed the experiments: MT and UB. Performed the experiments: MT and VP. Analyzed the data: MT, MW, FA, SH, SW, and UB. Contributed reagents/materials/analysis tools: SW, SH, and UB. Wrote the paper: MT and UB.

### Conflict of interest statement

The authors declare that the research was conducted in the absence of any commercial or financial relationships that could be construed as a potential conflict of interest.

## Supplementary material

Supplementary Figure S1 Anaerobic respiration in *P. aeruginosa*. ANR acts as the master oxygen-sensing regulator, which activates transcription of the *narXL* operon, encoding a two-component nitrate responsive two-component system. Under low-oxygen tension and in the presence of nitrate, ANR and NarL activate the transcription of the *narK1K2GHJI* operon encoding nitrate/nitrite transporters, and the structural genes for the respiratory nitrate reductase, which converts nitrate into nitrite. ANR and NarL co-operatively activate the *dnr* gene encoding the NO_2_/NO responsive regulator DNR. DNR activates the transcription of all other denitrification genes, encoding enzyme complexes that catalyze the conversion of nitrate into N_2_. NirQ requires DNR and NarL for its synthesis, and is predicted to be involved in fine tuning of the nitrite reductase activities. Nar, Nir, Nor, and Nos indicate nitrate reductase, nitrite reductase, nitric oxide reductase, and nitrous oxide reductase, respectively (adapted from Schreiber et al., 2007).

Supplementary Figure S2 **(A)** Genomic location of *paiI* and read coverage of the *paiI* transcript under conditions P, A-30 and B-96 visualized by the Genome Browser. **(B)** The transcriptional start site (TSS) of the *paiI* gene was determined by primer extension analysis with total RNA isolated from B-96 cells of PA14 (Lane 1). Sequence reactions (T, G, C, and A) were performed with the same primer using *in vitro* transcribed PaiI-1 (141 nt) sRNA as template and run in parallel. The transcriptional start (+1 A) is indicated on the left. The upstream and downstream sequences are shown on the right. F, primer extension signal obtained for the *in vitro* transcribed PaiI-1 RNA (141 nt). **(C)** Genetic locus encoding PaiI. The−10 and−35 promoter regions are colored in green, the transcriptional start site (TSS) and the NarL binding sites are show in red and violet, respectively. The consensus NarL signature (Schreiber et al., 2007) is shown boxed. The deleted NarL binding site within the *paiI* promoter sequence in strain PA14Δ*narL-paiI* is in violet. The numbers refer to the PA14 genome coordinates (http://www.pseudomonas.com).

Supplementary Figure S3 PaiI Secondary structure. **(A)** Predicted PaiI secondary structure according to *RNAfold* WebServer (http://rna.tbi.univie.ac.at/cgi-bin/RNAWebSuite/RNAfold.cgi). **(B)** Mapping of T1 cleavage sites in PaiI. Lane 1, alkaline ladder. Lanes 2, 3, and 4, P^32^ labeled PaiI was treated with 0.2 U of RNase T1 for 10 min, 15 min and 0 min respectively. Arrows depict the RNase T1 cleavage sites. Right, secondary structure of PaiI derived from the assay.

Supplementary Figure S4 Nitrate dependent synthesis of PaiI. Detection of PaiI by Northern-blotting in total RNA extracted from strain PA14 after 96 h of anoxic growth in BSM medium supplemented with 20 mM glucose. (+) and (–), presence and absence of nitrate in the medium, respectively.

Supplementary Figure S5 Nitrate reductase activity of strains PA14 and PA14Δ*paiI*. The nitrate reductase activity was determined in whole cell extracts of PA14 and PA14Δ*paiI*. The cultures were grown under the same conditions as described in the legend to Figure 4.

Supplementary Figure S6 **(A)** Putative base-pairing between PaiI and *nirS* mRNA. Putative secondary structure of the *nirS* mRNA upstream of the start codon predicted by Mfold (http://unafold.rna.albany.edu/?q=mfold/RNA-Folding-Form2.3). The AUG start codon is shown boxed and the Shine and Dalgarno sequence (SD) is indicated by a red bar. The putative PaiI interacting region is indicated by a blue bar. **(B)** PaiI and DNR-overproduction does not affect the NirS levels. The strains PA14, PA14Δ*paiI*, PA14Δ*paiI*(pMMBΔ*rbs*), and PA14Δ*paiI*(pMMB-*dnr*) were grown as described in the legend to Figure 4. At the indicated times after shift to anaerobiosis, samples were withdrawn and equal amounts of protein were separated on SDS-polyacrylamide gels. NirS was detected with anti-NirS antibodies. Ribosomal protein S2 served as a loading control. Lane M, molecular weight markers.
