# Supplementary figures and images for: The Anaerobically Induced sRNA PaiI Affects Denitrification in Pseudomonas aeruginosa PA14
Source: Front Microbiol. 2017 Nov 23;8:2312. doi: 10.3389/fmicb.2017.02312 (PMC5703892; doi:10.3389/fmicb.2017.02312)

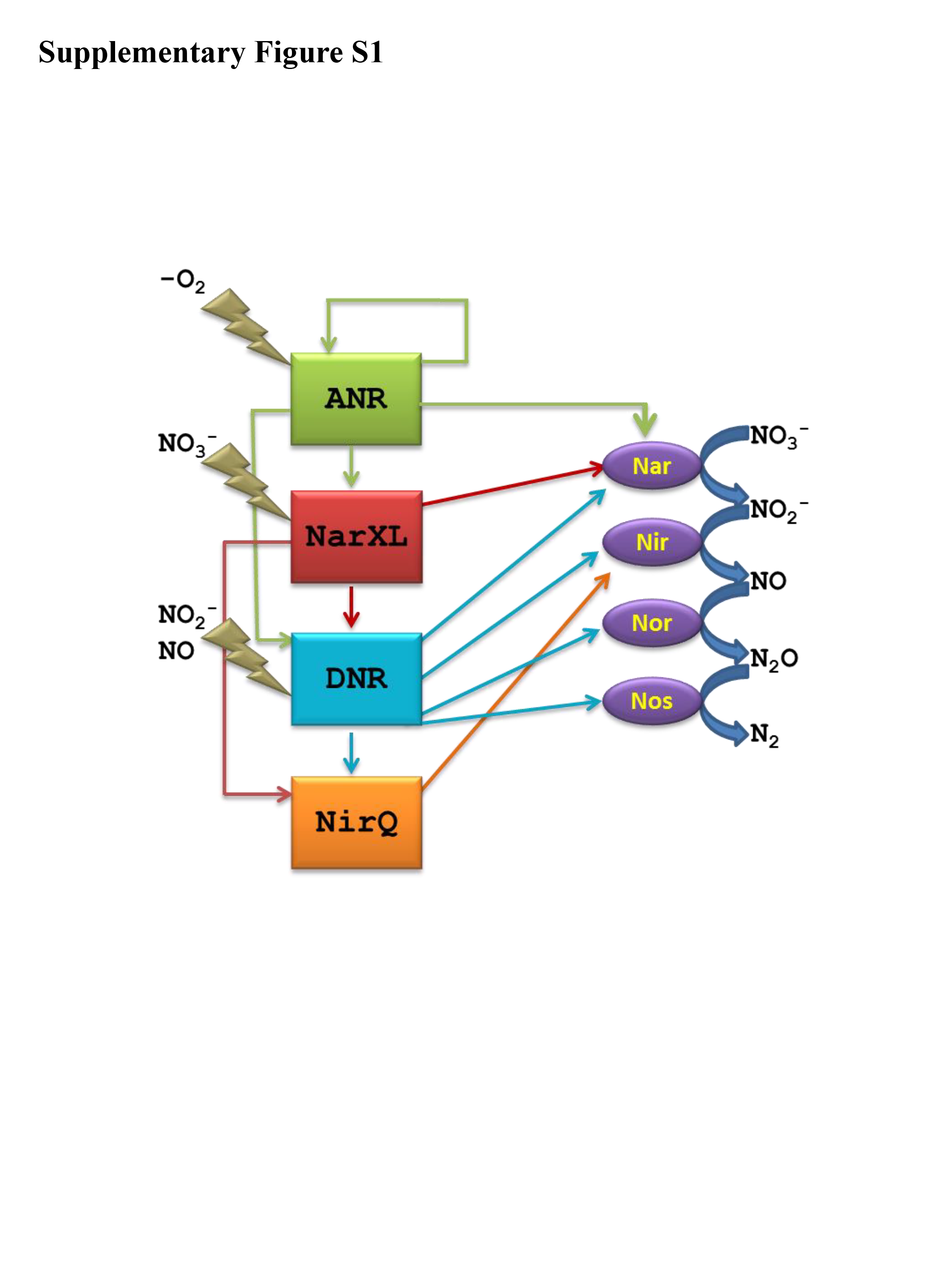

Supplement: Supplementary Figure S1 — Anaerobic respiration in P. aeruginosa. ANR acts as the master oxygen-sensing regulator, which activates transcription of the narXL operon, encoding a two-component nitrate responsive two-component system. Under low-oxygen tension and in the presence of nitrate, ANR and NarL activate the transcription of the narK1K2GHJI operon encoding nitrate/nitrite transporters, and the structural genes for the respiratory nitrate reductase, which converts nitrate into nitrite. ANR and NarL co-operatively activate the dnr gene encoding the NO2/NO responsive regulator DNR. DNR activates the transcription of all other denitrification genes, encoding enzyme complexes that catalyze the conversion of nitrate into N2. NirQ requires DNR and NarL for its synthesis, and is predicted to be involved in fine tuning of the nitrite reductase activities. Nar, Nir, Nor, and Nos indicate nitrate reductase, nitrite reductase, nitric oxide reductase, and nitrous oxide reductase, respectively (adapted from Schreiber et al., 2007). [file Image1.TIF]

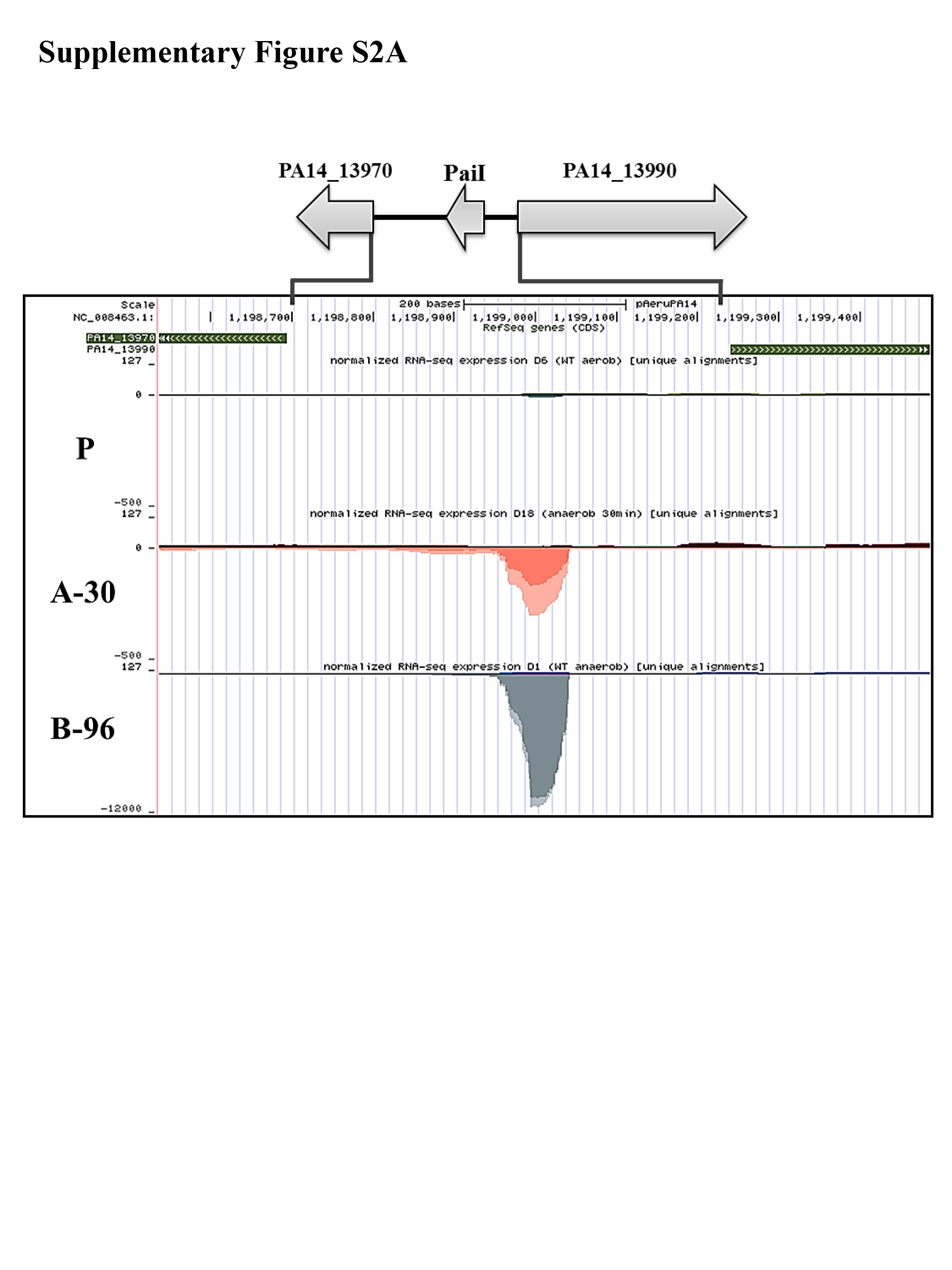

Supplement: Supplementary Figure S2 — (A) Genomic location of paiI and read coverage of the paiI transcript under conditions P, A-30 and B-96 visualized by the Genome Browser. (B) The transcriptional start site (TSS) of the paiI gene was determined by primer extension analysis with total RNA isolated from B-96 cells of PA14 (Lane 1). Sequence reactions (T, G, C, and A) were performed with the same primer using in vitro transcribed PaiI-1 (141 nt) sRNA as template and run in parallel. The transcriptional start (+1 A) is indicated on the left. The upstream and downstream sequences are shown on the right. F, primer extension signal obtained for the in vitro transcribed PaiI-1 RNA (141 nt). (C) Genetic locus encoding PaiI. The−10 and−35 promoter regions are colored in green, the transcriptional start site (TSS) and the NarL binding sites are show in red and violet, respectively. The consensus NarL signature (Schreiber et al., 2007) is shown boxed. The deleted NarL binding site within the paiI promoter sequence in strain PA14ΔnarL-paiI is in violet. The numbers refer to the PA14 genome coordinates (http://www.pseudomonas.com). [file Image2.TIF]

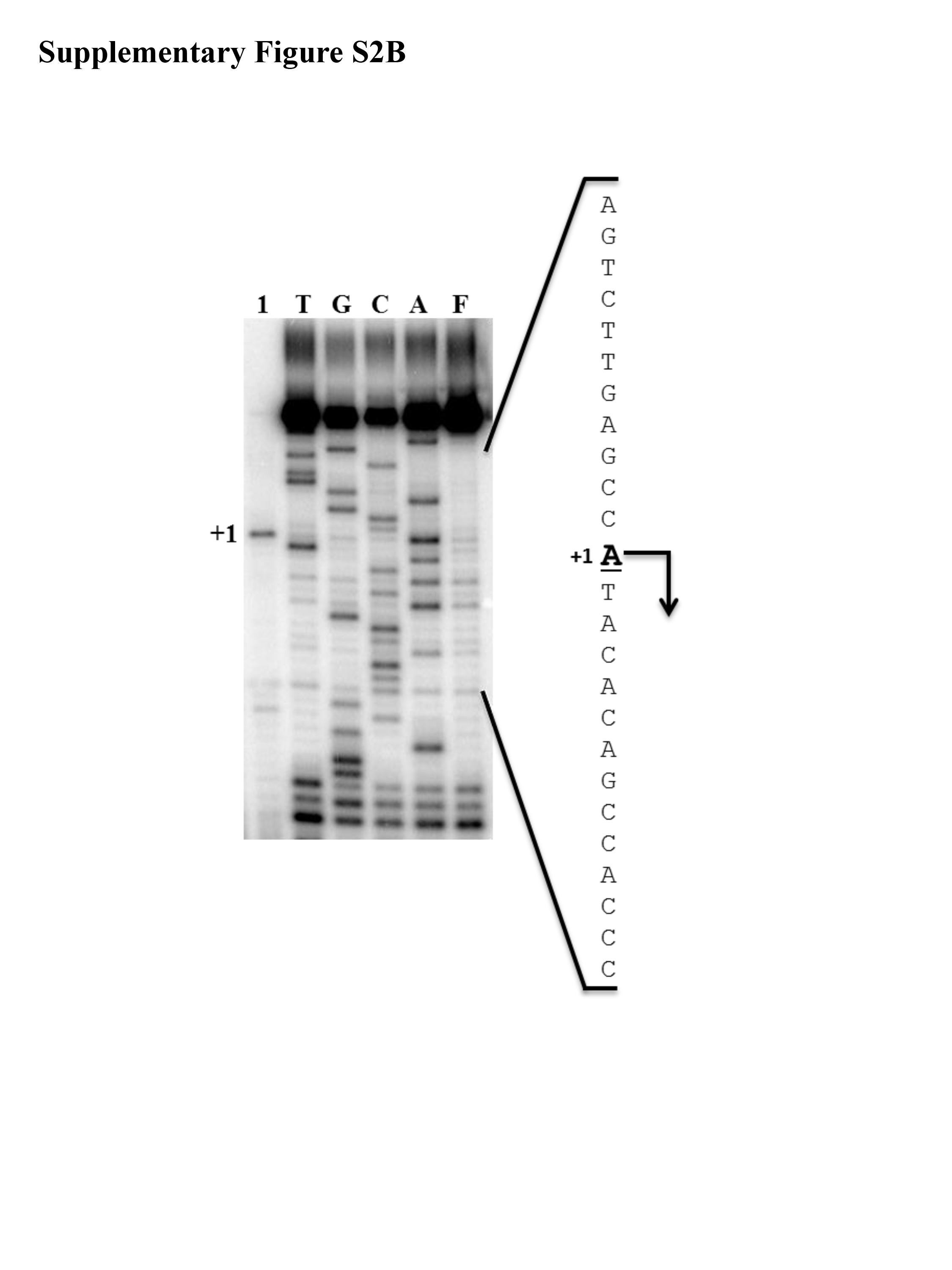

Supplement: Supplementary file 3 [file Image3.TIF]

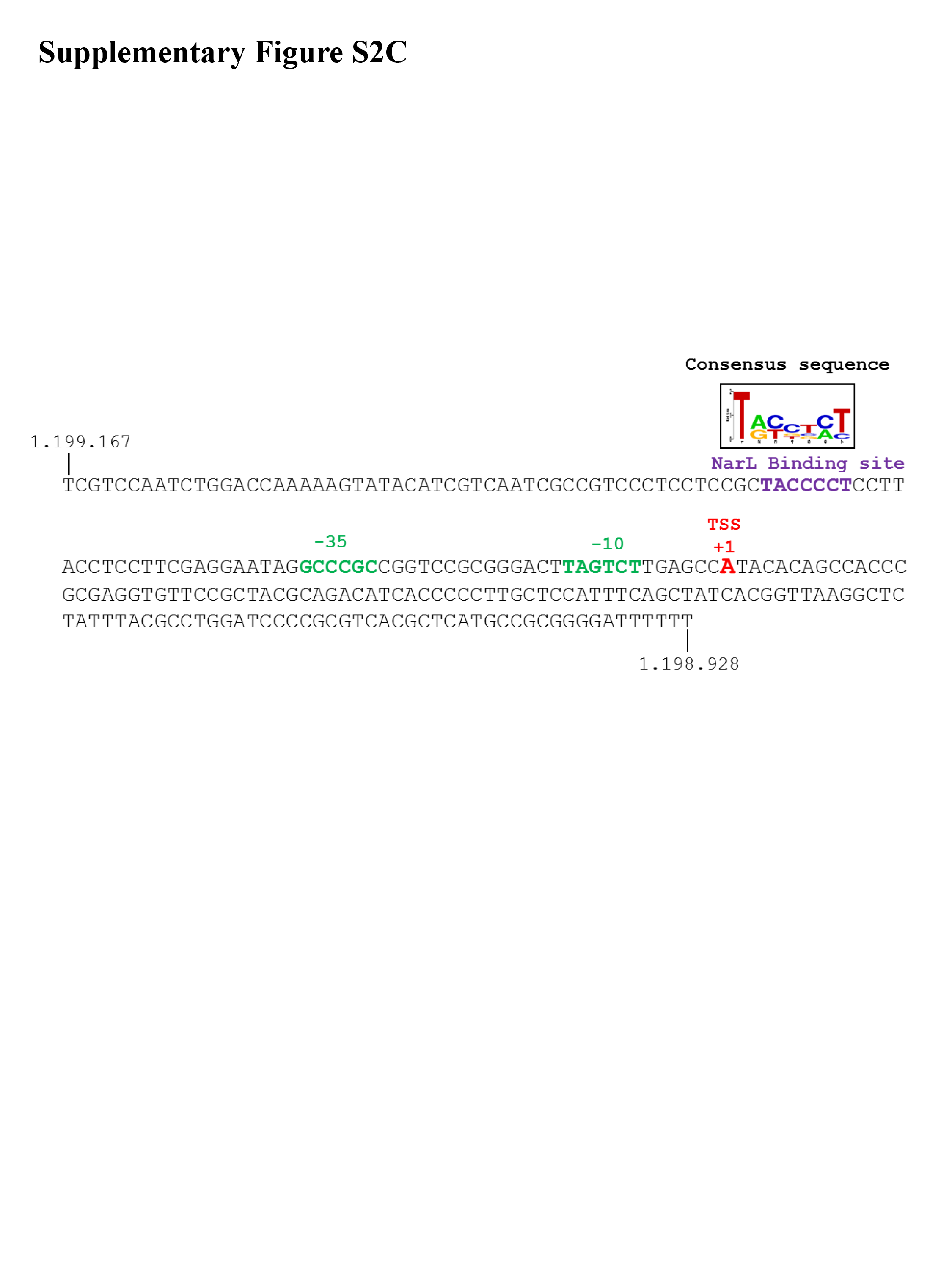

Supplement: Supplementary file 4 [file Image4.TIF]

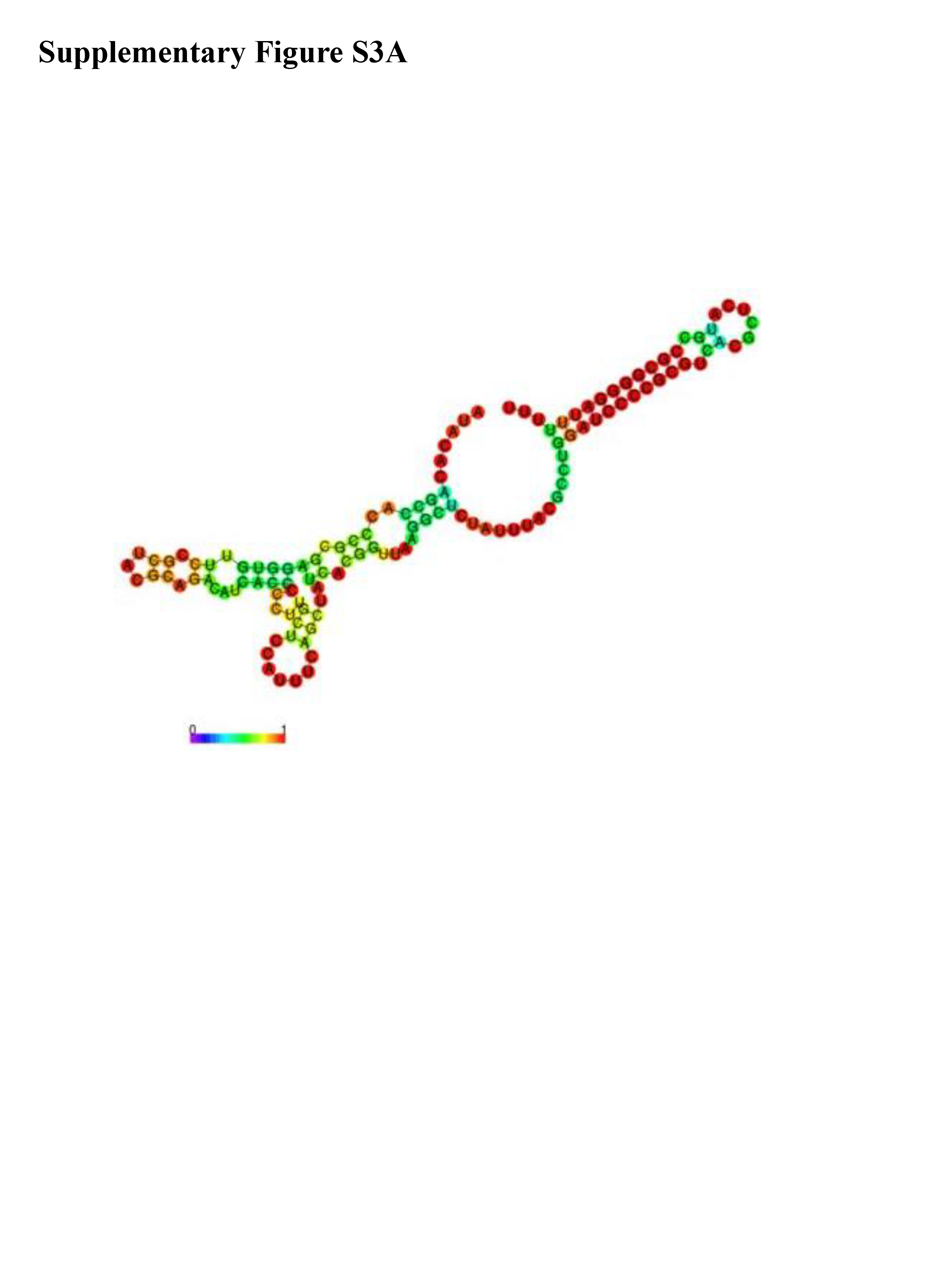

Supplement: Supplementary Figure S3 — PaiI Secondary structure. (A) Predicted PaiI secondary structure according to RNAfold WebServer (http://rna.tbi.univie.ac.at/cgi-bin/RNAWebSuite/RNAfold.cgi). (B) Mapping of T1 cleavage sites in PaiI. Lane 1, alkaline ladder. Lanes 2, 3, and 4, P32 labeled PaiI was treated with 0.2 U of RNase T1 for 10 min, 15 min and 0 min respectively. Arrows depict the RNase T1 cleavage sites. Right, secondary structure of PaiI derived from the assay. [file Image5.TIF]

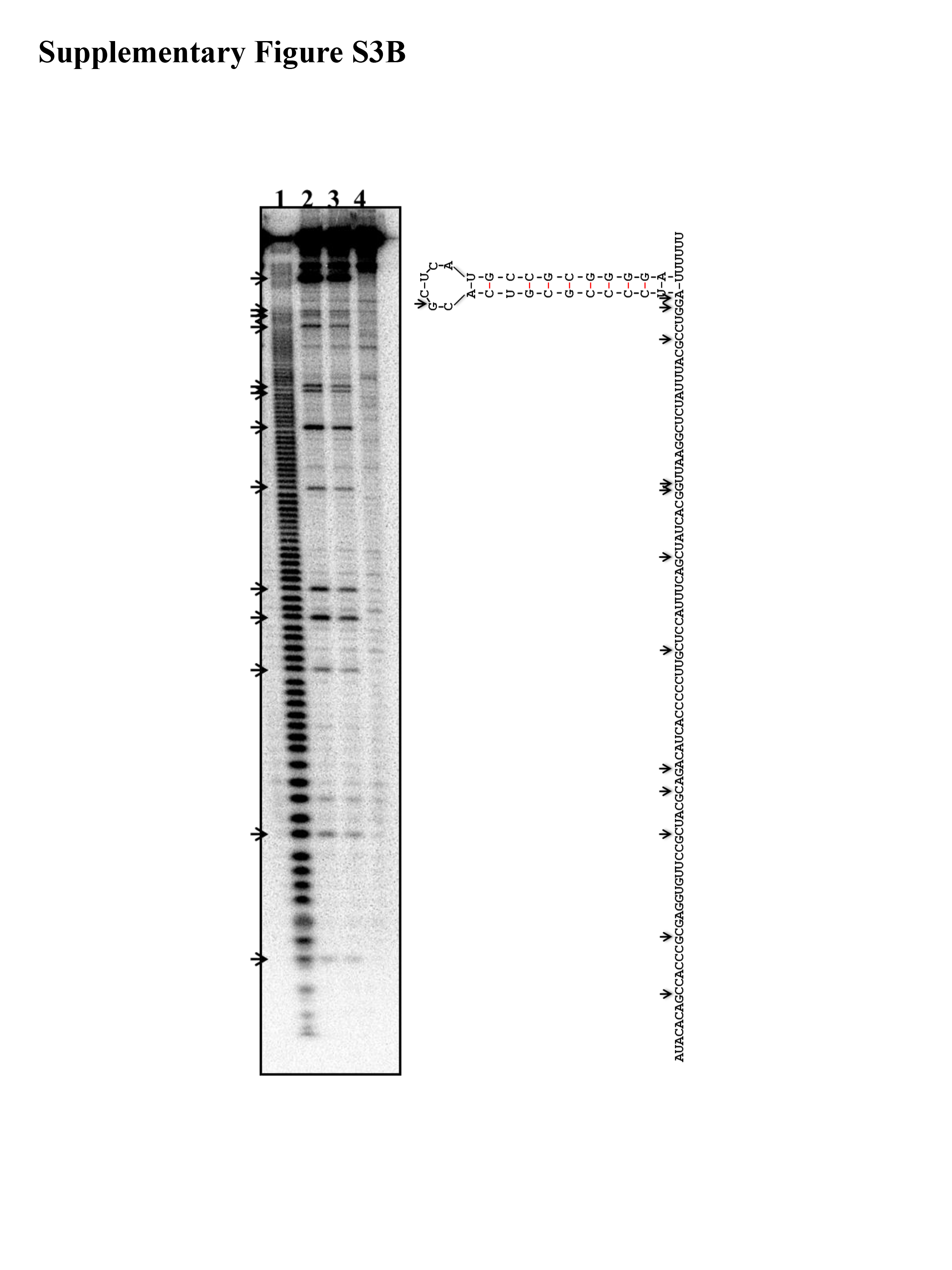

Supplement: Supplementary file 6 [file Image6.TIF]

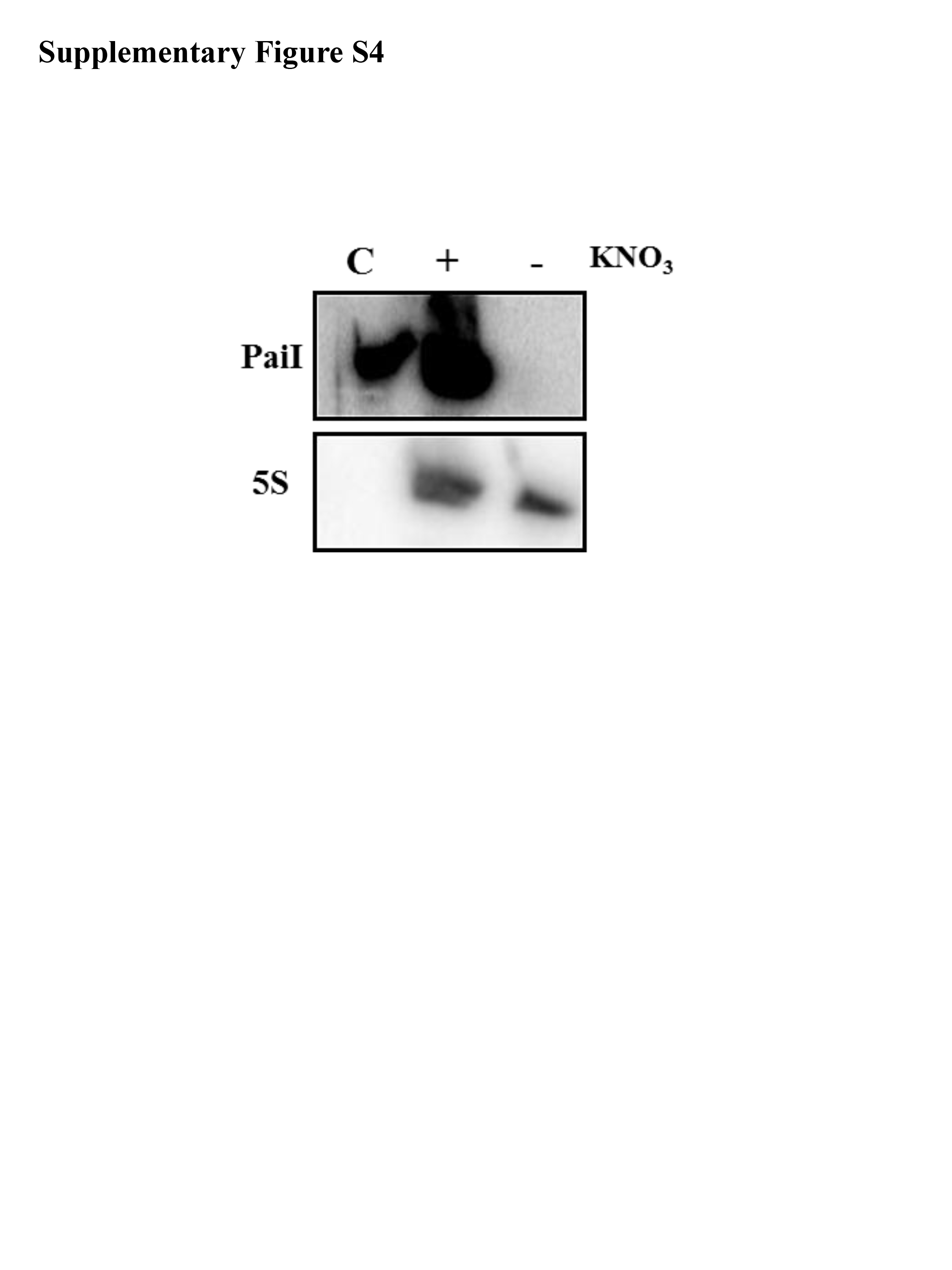

Supplement: Supplementary Figure S4 — Nitrate dependent synthesis of PaiI. Detection of PaiI by Northern-blotting in total RNA extracted from strain PA14 after 96 h of anoxic growth in BSM medium supplemented with 20 mM glucose. (+) and (–), presence and absence of nitrate in the medium, respectively. [file Image7.TIF]

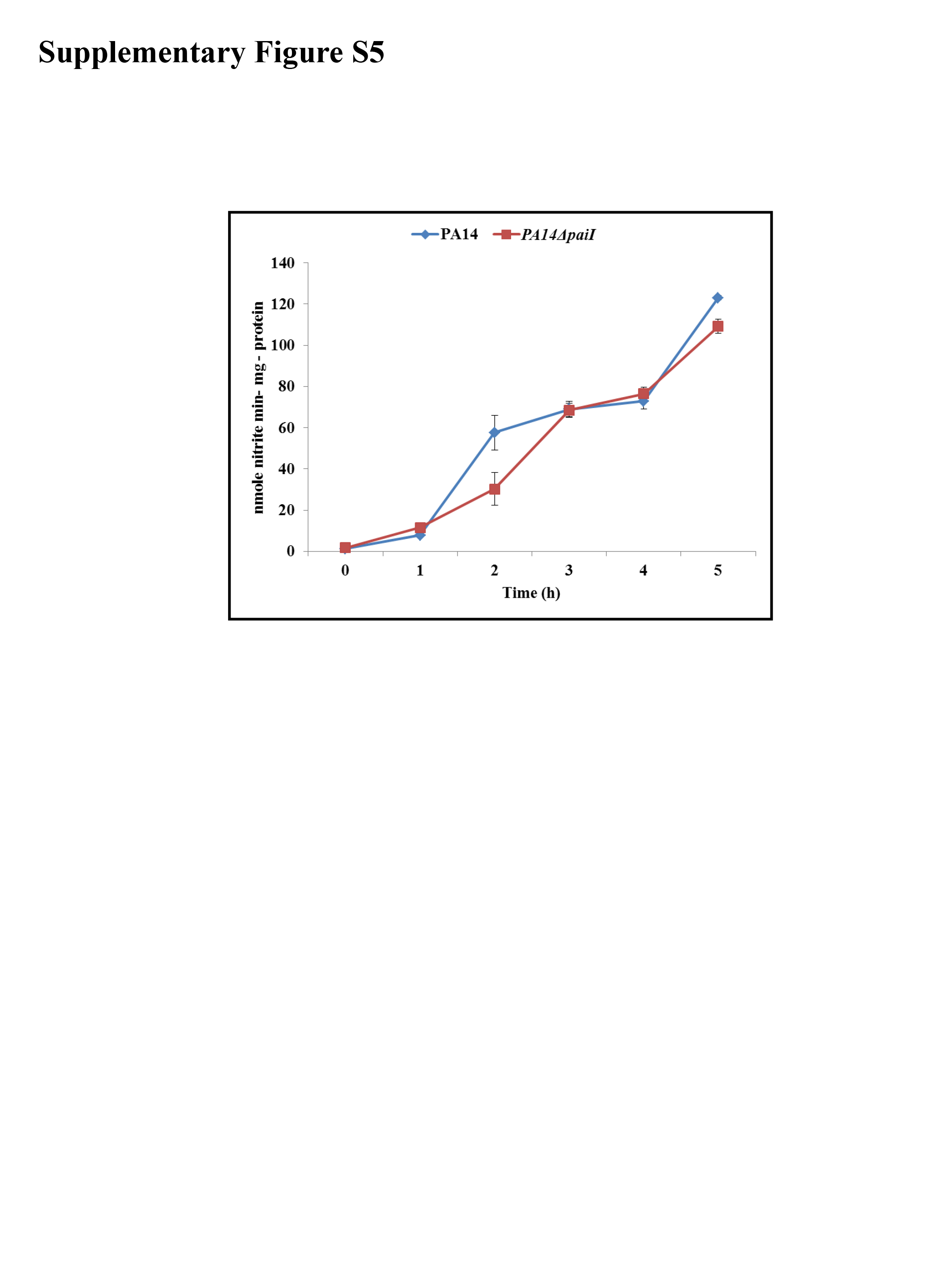

Supplement: Supplementary Figure S5 — Nitrate reductase activity of strains PA14 and PA14ΔpaiI. The nitrate reductase activity was determined in whole cell extracts of PA14 and PA14ΔpaiI. The cultures were grown under the same conditions as described in the legend to Figure 4. [file Image8.TIF]

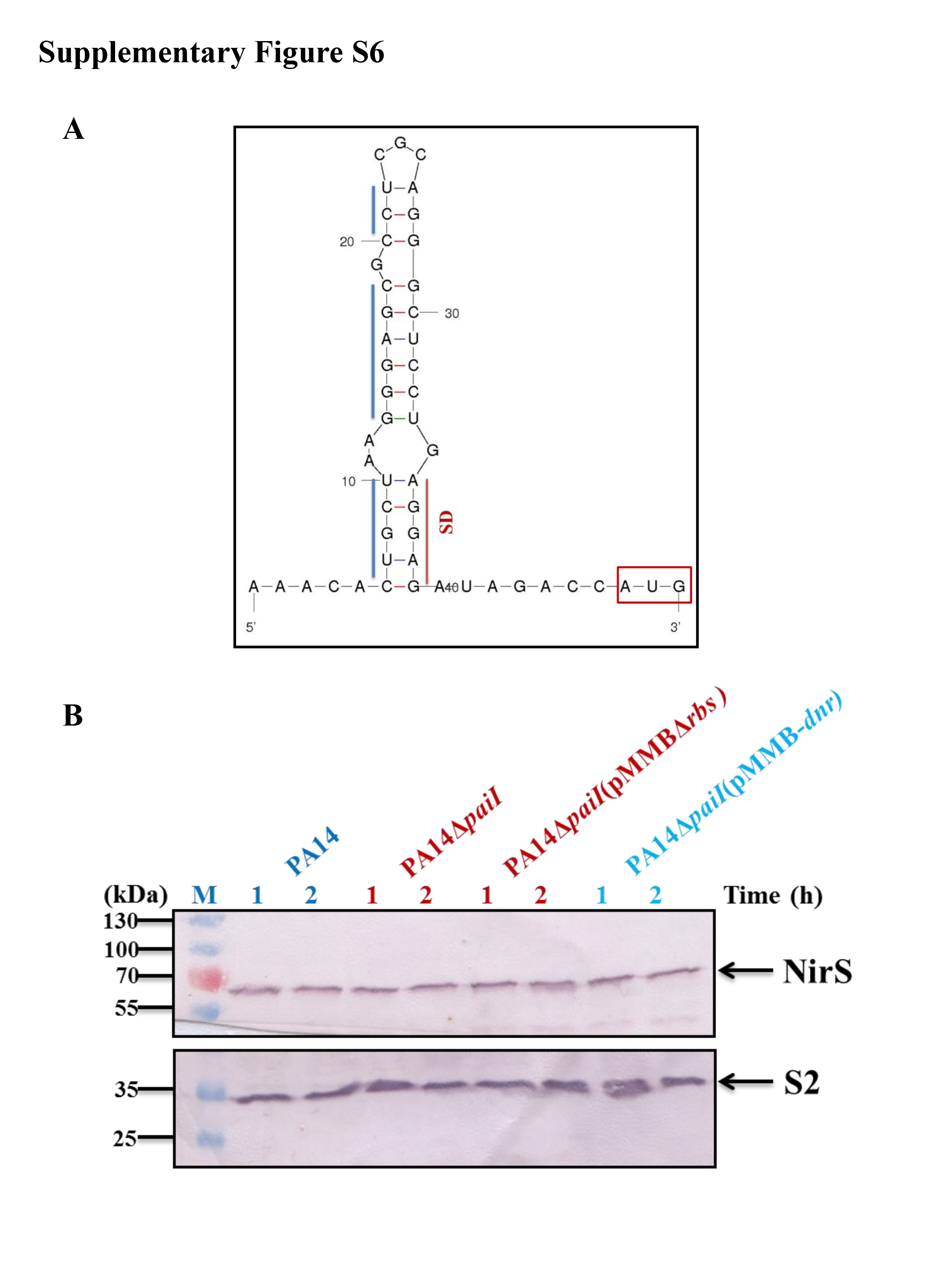

Supplement: Supplementary Figure S6 — (A) Putative base-pairing between PaiI and nirS mRNA. Putative secondary structure of the nirS mRNA upstream of the start codon predicted by Mfold (http://unafold.rna.albany.edu/?q=mfold/RNA-Folding-Form2.3). The AUG start codon is shown boxed and the Shine and Dalgarno sequence (SD) is indicated by a red bar. The putative PaiI interacting region is indicated by a blue bar. (B) PaiI and DNR-overproduction does not affect the NirS levels. The strains PA14, PA14ΔpaiI, PA14ΔpaiI(pMMBΔrbs), and PA14ΔpaiI(pMMB-dnr) were grown as described in the legend to Figure 4. At the indicated times after shift to anaerobiosis, samples were withdrawn and equal amounts of protein were separated on SDS-polyacrylamide gels. NirS was detected with anti-NirS antibodies. Ribosomal protein S2 served as a loading control. Lane M, molecular weight markers. [file Image9.TIF]
