# Supplementary material for: The Anaerobically Induced sRNA PaiI Affects Denitrification in Pseudomonas aeruginosa PA14
Source: Front Microbiol. 2017 Nov 23;8:2312. doi: 10.3389/fmicb.2017.02312 (PMC5703892; doi:10.3389/fmicb.2017.02312)
Supplement: Supplementary file 10 [file Table1.docx]

**Supplementary Table S1.**  Strains and plasmids used in this study.

| **Strain/plasmid** | **Genotype/relevant features** | **Source/reference** |
| --- | --- | --- |
| ***P. aeruginosa*** |  |  |
| PA14 |  | (Liberati *et al.*, 2006) |
| PAO1 |  | (Holloway *et al.*, 1979) |
| PAO6261 | PAO1Δ*anr* | (Ye *et al.*, 1995) |
| PAO9104 | PAO1Δ*narL* | (Schreiber *et al.*, 2007) |
| PA14Δ*paiI* | In-frame deletion of *paiI* in strain PA14 | This study |
| PA14Δ*narL-paiI* | Deletion of NarL Binding site within the *paiI* promoter sequence in strain PA14 | This study |
|  |  |  |
| ***E. coli*** |  |  |
| DH5α | *recA1 endA1 hsdR17 thi-1 supE44 gyrA96 relA1 deoR*  *Δ(lacZYA-argF) U169 (Φ80lacZΔM15)* | (Sambrook and Russel, 2001) |
| HB101 | *thi-1 hsdS20(rB-, mB-) supE44 recA13 ara-14 leuB6 proA2 lacY1 galK2 xyl-5 mtl-1 rpsL20* | (Sambrook and Russel, 2001) |
|  |  |  |
| **Plasmids** |  |  |
| pME4510 | Broad–host-range promoter-probe plasmid, Gm^r^ | (Rist and Kertesz, 1998) |
| pME4510-1 | pME4510 carrying *lacI^Q^* and *tac* promoter | This study |
| pME-*paiI* | pME4510-1 harboring *paiI* under transcriptional control of P*_tac_* | This study |
| pMMBΔ*rbs* | pMMB67HE with a deletion of the RBS of *lacZ* | (Sonnleitner and Bläsi, 2014) |
| pMMB-*anr* | pMMBΔ*rbs* carrying *anr* under transcriptional control of P*_tac_* | This study |
| pMMB-*dnr* | pMMBΔ*rbs* carrying *dnr*  under transcriptional control of P*_tac_* | This study |
| pMMB-*nirQ* | pMMBΔ*rbs* carrying *nirQ* under transcriptional control of P*_tac_* | This study |
| pME3087 | Suicide vector, ColE1 replicon, IncP-1, Mob; Tc^r^ | (Voisard *et al.*, 2007) |
| pRK2013 | Helper plasmid, ColE1 replicon, Tra; Km^r^ | (Figurski and Helinski, 1979) |
