# Supplementary material for: The Anaerobically Induced sRNA PaiI Affects Denitrification in Pseudomonas aeruginosa PA14
Source: Front Microbiol. 2017 Nov 23;8:2312. doi: 10.3389/fmicb.2017.02312 (PMC5703892; doi:10.3389/fmicb.2017.02312)
Supplement: Supplementary file 11 [file Table2.DOCX]

**Supplementary Table S2**. **Oligonucleotides used in this study.** Restriction sites are highlighted in bold and are in italics.

| **Name** | **Sequence (5' → 3')** | **Description** |
| --- | --- | --- |
| G84 | GCGGAACACCTCGCGGGTGGC | Probe for detection of PaiI |
| J126 | gata***aagctt***ggaattgtgagcggataacaatttcacacaggaaacagaatATGGCCGAAACCATCAAGG | Forward primer for cloning of *anr* |
| K126 | TGAT***GGATCC***TCAGCCTTCCAGCTGGCCG | Reverse primer for cloning of *anr* |
| N126 | gata***aagctt***ggaattgtgagcggataacaatttcacacaggaaacagaatATGAACAAGGCGACCGCGCC | Forward primer for cloning of *dnr* |
| O126 | TGAT***GGATCC***TTACACCAGGTCGTTCTCCAC | Reverse primer for cloning of *dnr* |
| P126 | gata***aagctt***ggaattgtgagcggataacaatttcacacaggaaacagaatATGCGGGACGCGACACCCTTC | Forward primer for cloning of *nirQ* |
| Q126 | TGAT***GGATCC***TCAGGCGACATGGAGATCGAC | Reverse primer for cloning of *nirQ* |
| I26 | CCCCACACTACCATCGGCGATGCGTCG | Probe for detection of 5S rRNA |
| C99 | GCATAC***GGTACC***ATTGCCTCCAGAAAATAAAGG | Deletion of *paiI* in PA14 |
| D99 | GCTCAT***TCTAGA***TCCTGGCTGGTGTAGCGCACC | Deletion of *paiI* in PA14 |
| E99 | TCCCCGCGTCACGCTCATGCCGCGG | Deletion of *paiI* in PA14 |
| F99 | CCGCGGCATGAGCGTGACGCGGGGAGTGTATGGCTCAAGACTAAGTCCCGCG | Deletion of *paiI* in PA14 |
| H109 | TAATACGACTCACTATAGGATACACAGCCACCCGCGAG | Forward primer to generate template for *in vitro* transcription of PaiI |
| I86 | AAAAAATCCCCGCGGCATGAGCGTG | Reverse primer to generate template for *in vitro* transcription of PaiI |
| Q106 | TAATACGACTCACTATAGGTAGTCTTGAGCCATACACAGC | Forward primer to generate template for *in vitro* transcription of PaiI-1 used for primer extension analysis |
| R105 | CCGTGATAGCTGAAATGGAGC | Primer extension analysis to map the transcriptional start site of *paiI* |
| P107 | GATA***CCCGGG***ATACACAGCCACCCGCGAGGTG | Cloning of *paiI* into pME4510-1 |
| H99 | ACGT***CTGCAG***AAAAAATCCCCGCGGCATGAGCGTG | Cloning of *paiI* into pME4510-1 |
| L85 | GATATC***GAATTC***GAACGCCAGCAAGAC | Forward primer for construction of pME4510-1 |
| Q107 | GATATC***CCCGGG***ATTATATTGTTATCCGCTCACAATGTCAATTGTTATCCGCTCACAATTCAGAATATTTGCCAGAACCG | Reverse primer for construction of pME4510-1 |
| P105 | CTTACCTCCTTCGAGGAATAG | Deletion of the NarL binding site within the *paiI* gene promoter sequence |
| Q105 | CTATTCCTCGAAGGAGGTAAGCGGAGGAGGGACGGCGATTGACG | Deletion of the NarL binding site within the *paiI* gene promoter sequence |
