# Supplementary material for: The Anaerobically Induced sRNA PaiI Affects Denitrification in Pseudomonas aeruginosa PA14
Source: Front Microbiol. 2017 Nov 23;8:2312. doi: 10.3389/fmicb.2017.02312 (PMC5703892; doi:10.3389/fmicb.2017.02312)
Supplement: Supplementary file 12 [file Table3.DOCX]

**Supplementary Table S3. Small RNAs that are differentially abundant in A-30 and B-96 cells when compared with P cells.** The log2-fold change refers to the abundance of transcripts in A-30 and B-96 cells when compared with P cells. Only transcripts that showed ≥ ± 1.5 log2-fold change and a p-value ≤0.05 were considered to be significant.

|  | **A-30 vs P** |  | **B-96 vs P** |  |  | |  |  |
| --- | --- | --- | --- | --- | --- | --- | --- | --- |
|  | **Log2-fold Change** | p-value | **Log2-fold Change** | **p-value** | **Start** | **Stop** | **Note** | **Reference** |
| PA14_13970.1 | 2.559 | 1.31E-28 | 7.993 | 2.93E-291 | 1198694 | 1200056 | H/N/NB |  |
| P24 | 1.355 | 1.99E-03 | 3.954 | 2.14E-24 | 1080027 | 1080408 | NB | Livny *et al.,* 2006 |
| PA14_36030.1 | 1.381 | 8.03E-02 | 2.995 | 7.16E-20 | 3208643 | 3209913 | N |  |
| PA14_53820.1 | 1.568 | 2.61E-26 | 2.865 | 1.19E-44 | 4771227 | 4774170 |  |  |
| SPA0115 | -1.089 | 1.45E-09 | 2.648 | 4.23E-24 | 2453901 | 2454790 | A/NB | Ferrara *et al.*, 2012 |
| PA14_47800.1 | 2.222 | 4.94E-26 | 2.275 | 5.44E-18 | 4252792 | 4253143 | N |  |
| PA14sr_090 | -1.303 | 4.16E-17 | 2.007 | 8.78E-16 | 3601180 | 3601463 | H | Wurtzel *et al.*, 2012 |
| PA14_61250.1 | 2.002 | 2.42E-23 | 1.801 | 8.69E-12 | 5468812 | 5469164 |  |  |
| SPA0147 | 0.819 | 1.72E-07 | 1.712 | 8.40E-17 | 2236447 | 2239720 | H/N | Ferrara *et al.*, 2012 |
| SPA0011 | -0.765 | 8.85E-06 | 1.589 | 4.36E-11 | 2677544 | 2682979 | H/N/NB | Ferrara *et al.*, 2012 |
| SPA0105 | -4.136 | 1.25E-170 | 1.216 | 5.27E-09 | 1648208 | 1648631 | H | Ferrara *et al.*, 2012 |
| SPA0104 | -3.758 | 2.43E-112 | -1.398 | 1.10E-10 | 1651715 | 1651895 | H/NB | Ferrara *et al.*, 2012 |
| SPA0012 | -2.917 | 2.98E-59 | -1.405 | 2.15E-09 | 3515246 | 3522477 | H/NB | Ferrara *et al.*, 2012 |
| PA14sr_006 | -1.158 | 5.36E-11 | -1.951 | 9.77E-17 | 181429 | 181627 |  | Wurtzel *et al.*, 2012 |
| PA14_28350.1 | -6.314 | 5.88E-169 | -2.316 | 2.42E-12 | 2450374 | 2452095 |  |  |
| PA14_68290.1 | 0.056 | 7.22E-01 | -2.355 | 1.12E-24 | 6091008 | 6093085 |  |  |
| PA14_47210.1 | -1.941 | 3.49E-31 | -2.524 | 9.92E-31 | 4206553 | 4207179 |  |  |
| SPA0133 | 0.282 | 2.03E-01 | -2.637 | 1.17E-18 | 2348175 | 2348300 |  | Ferrara *et al.*, 2012 |
| PA14_11660.1 | -3.531 | 2.10E-107 | -2.845 | 4.48E-37 | 1011554 | 1011900 |  |  |
| P34 | -1.753 | 3.22E-33 | -3.109 | 2.58E-51 | 6102964 | 6108683 | NB | Livny *et al.,* 2006 |

H: Interacts with Hfq (Pusic *et al.,* 2016), A: Putative Anr binding site, N: Putative NarL binding site, NB: Detected by Northern-blotting.
