# Supplementary material for: The Anaerobically Induced sRNA PaiI Affects Denitrification in Pseudomonas aeruginosa PA14
Source: Front Microbiol. 2017 Nov 23;8:2312. doi: 10.3389/fmicb.2017.02312 (PMC5703892; doi:10.3389/fmicb.2017.02312)
Supplement: Supplementary file 13 [file Table4.DOCX]

| **PA14-ID** | **Gene** | **Fold change**  **PA14**  **vs**  **PA14*ΔpaiI*** | **TPM PA14** | **TPM PA14*ΔpaiI*** | **p-value** | **Function** |
| --- | --- | --- | --- | --- | --- | --- |
| PA14_23680 | *ibpA* | 2.078 | 485.157 | 196.946 | 1.63E-26 | heat-shock protein IbpA |
| PA14_03650 | *cysA* | 1.823 | 76.159 | 22.860 | 4.15E-05 | sulfate transport protein CysA |
| PA14_40780 |  | 1.807 | 66.549 | 28.391 | 4.34E-07 | hypothetical protein |
| PA14_22080 |  | 1.731 | 76.303 | 34.034 | 2.35E-06 | resolvase |
| PA14_09540 | *mexG* | 1.715 | 63.574 | 31.263 | 2.74E-12 | hypothetical protein |
| PA14_15360 |  | 1.691 | 52.610 | 20.305 | 2.06E-04 | hypothetical protein |
| PA14_40770 | *cysI* | 1.659 | 59.124 | 29.229 | 6.46E-07 | sulfite reductase |
| PA14_57710 | *cysN* | 1.617 | 154.319 | 76.127 | 2.60E-05 | bifunctional sulfate adenylyltransferase subunit 1/adenylylsulfate kinase |
| PA14_09530 | *mexH* | 1.598 | 50.264 | 26.559 | 1.11E-08 | RND efflux membrane fusion protein |
| PA14_13580 |  | 1.592 | 98.044 | 46.811 | 3.02E-04 | ABC transporter ATP-binding protein |
| PA14_36200 |  | 1.581 | 114.096 | 57.619 | 6.60E-05 | ABC transporter substrate-binding protein |
| PA14_61840 | *vapI* | 1.567 | 57.305 | 28.722 | 1.41E-04 | virulence-associated protein |
| PA14_52570 | *rsmA* | 1.558 | 310.392 | 151.430 | 4.53E-04 | carbon storage regulator |
| PA14_57020 | *groES* | 1.535 | 346.938 | 181.778 | 9.65E-05 | co-chaperonin GroES |
| PA14_19680 |  | 1.515 | 61.015 | 33.702 | 1.49E-05 | hypothetical protein |
| PA14_11670 |  | -1.598 | 26.451 | 49.252 | 1.05E-03 | hypothetical protein |
| PA14_11660 | *aqpZ* | -1.768 | 118.087 | 210.996 | 1.11E-06 | aquaporin Z |
| PA14_13970 |  | -2.100 | 17.703 | 48.436 | 1.04E-07 | hypothetical protein |
| PA14_13960 |  | -2.420 | 23.151 | 68.186 | 8.23E-12 | hypothetical protein |

**Supplementary Table S4.** T**ranscripts that are differentially abundant in PA14 when compared with PA14*ΔpaiI*.** The fold-change refers to the abundance of transcripts in PA14 when compared with PA14*ΔpaiI*. Only transcripts with a fold change ≥ ± 1.5, a p-value ≤ 0.05 and Transcripts Per Million reads (TPM) ≥ 50 in at least one strain were considered. Genes are ordered according to fold change (http://www.pseudomonas.com).
